# Dynamical Correlations Reveal Allosteric Sites in G Protein-Coupled Receptors

**DOI:** 10.3390/ijms22010187

**Published:** 2020-12-27

**Authors:** Pedro Renault, Jesús Giraldo

**Affiliations:** 1 Laboratory of Molecular Neuropharmacology and Bioinformatics, Unitat de Bioestadística and Institut de Neurociències, Universitat Autònoma de Barcelona, 08193 Bellaterra, Spain; Pedro.Renault@uab.es; 2 Unitat de Neurociència Traslacional, Parc Taulí Hospital Universitari, Institut d’Investigació i Innovació Parc Taulí (I3PT), Institut de Neurociències, Universitat Autònoma de Barcelona, 08193 Bellaterra, Spain; 3 Instituto de Salud Carlos III, Centro de Investigación Biomédica en Red de Salud Mental, CIBERSAM, 08193 Bellaterra, Spain

**Keywords:** allosteric sites, GPCR, dynamical correlations, normal modes, coupled motions, molecular dynamics

## Abstract

G protein-coupled Receptors (GPCRs) play a central role in many physiological processes and, consequently, constitute important drug targets. In particular, the search for allosteric drugs has recently drawn attention, since they could be more selective and lead to fewer side effects. Accordingly, computational tools have been used to estimate the druggability of allosteric sites in these receptors. In spite of many successful results, the problem is still challenging, particularly the prediction of hydrophobic sites in the interface between the protein and the membrane. In this work, we propose a complementary approach, based on dynamical correlations. Our basic hypothesis was that allosteric sites are strongly coupled to regions of the receptor that undergo important conformational changes upon activation. Therefore, using ensembles of experimental structures, normal mode analysis and molecular dynamics simulations we calculated correlations between internal fluctuations of different sites and a collective variable describing the activation state of the receptor. Then, we ranked the sites based on the strength of their coupling to the collective dynamics. In the β2 adrenergic (β2AR), glucagon (GCGR) and M2 muscarinic receptors, this procedure allowed us to correctly identify known allosteric sites, suggesting it has predictive value. Our results indicate that this dynamics-based approach can be a complementary tool to the existing toolbox to characterize allosteric sites in GPCRs.

## 1. Introduction

G protein-coupled receptors (GPCRs) are membrane proteins that mediate cellular responses to a wide variety of signals, including hormones, neurotransmitters and drugs [1]. Their central role in physiology and in the pathophysiology of various diseases makes them key pharmacological targets [2,3]. Experimental data and computational simulations indicate that GPCR dynamics is essential for the performance of their biological functions [4,5,6]. The design of safer and more effective drugs must therefore take into account the motions that underlie the functional conformational changes of these receptors [7].

In the search for more selective drugs with fewer side effects, the investigation of allosteric sites in GPCRs has aroused growing interest [8,9]. Being less conserved than orthosteric sites, they could provide greater selectivity and finer control over the dynamical equilibrium of the receptor. Allosteric ligands could contribute to stabilize inactive or active conformations, or states potentially linked to biased signaling or partial agonism, thereby modulating the biological response [10,11,12].

New experimental structures of GPCRs with bound allosteric modulators have fostered the investigation of allosteric sites. In a recent study, FTMap and FTSite softwares were used to identify allosteric sites in the structures of 17 distinct GPCRs obtained in the presence of allosteric ligands [13]. These programs estimate druggability by mapping the protein with organic probes, which cluster around binding hot spots [14,15,16]. The predictions were successful in most cases, but failed at hydrophobic sites located at the protein-membrane interface, as in the case of the glucagon receptor (GCGR). These results highlight the difficulties of determining druggabilities in GPCRs and suggest the convenience of complementary approaches in the search for allosteric sites in these receptors.

Computational analysis of protein dynamics represents one such alternative. For instance, the PARS [17] and SPACER [18] programs calculate the effect of the perturbation of pockets on the vibrational normal modes of a protein, identifying as potential allosteric sites those leading to significant changes in the collective dynamics when perturbed. Other methods measure correlations between coordinates [19,20] or volumes [21] of distinct sites, assuming that allosteric sites are dynamically coupled to the regions they regulate. Although these approaches may provide insights into mechanisms of allosteric modulation, and in spite of the importance of collective motions for GPCR function, a detailed account of the dynamical couplings of binding sites in GPCRs is still lacking.

**Figure 1:**
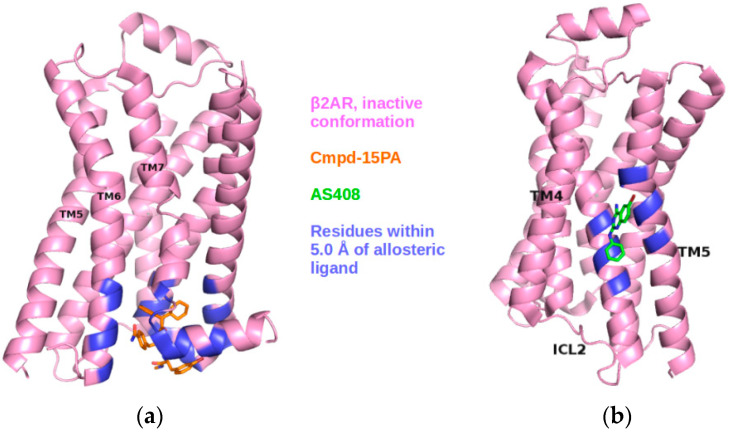

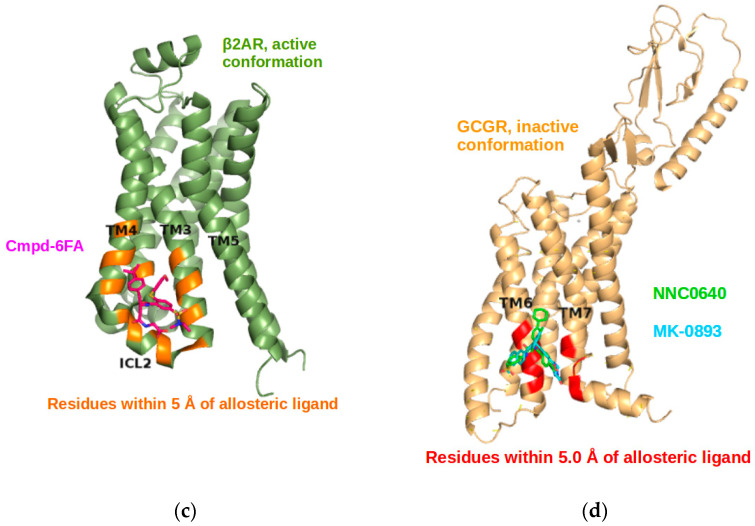
Experimental structures of β2AR and glucagon receptor (GCGR) in the presence of allosteric modulators. β2AR in the inactive conformation (pink) bound to (**a**) Cmpd-15PA (PDB: 5X7D); (**b**) AS408 (PDB: 6OBA; (**c**) β2AR in the active conformation (green), bound to Cmpd-6FA (PDB: 6N48); (**d**) GCGR in the inactive conformation, in the presence of the allosteric ligands NNC0640 and MK-0893 (green and cyan; PDB codes 5XEZ and 5EE7, respectively). Ligands are shown as sticks and residues within 5.0 Å of a ligand are highlighted in different colors.

In this work, we used computational tools to study the coupling of binding sites in GPCRs to the receptor’s collective motions and whether these couplings can reveal allosteric sites. Our initial hypotheses were that allosteric sites are strongly coupled with regions of the receptor which undergo significant conformational rearrangements upon activation, and that they can be distinguished from other cavities based on the strength of this coupling. As a representative case study, we investigated the β2 adrenergic receptor (β2AR), a prototypical class A GPCR. Three structures of the β2AR in the presence of allosteric modulators have recently been determined [22,23,24] (Figure 1a–c). Using experimental structures and also ensembles generated by normal mode analysis (NMA) and molecular dynamics (MD) simulations, we verified that allosteric sites in this receptor were correctly identified based on dynamical correlations. We also studied GCGR, an example of a class B GPCR. The dynamical analysis successfully spotted its known allosteric site [25,26] (Figure 1d), whose prediction had proven to be challenging [13]. In addition, we detected the known allosteric site on the M2 muscarinic receptor, another class A GPCR (Figure S1). Despite some limitations of the approach, discussed below, our results suggest that it can constitute a useful complementary tool in the characterization of allosteric sites in GPCRs.

## 2. Results

Our prediction of allosteric sites was based on correlations between inter-residue distance fluctuations. As detailed below, we measured the correlation between each pairwise distance with a collective variable describing the conformational state of the receptor. A distance highly correlated with this variable indicated the coupling of that residue pair to the collective motion of activation. In particular, if distances highly correlated to the activation motion were found inside a binding site, this pointed to the importance of this site for the global dynamics of the receptor and to its possible role in the allosteric modulation of conformational changes.

### 2.1. The Collective Variable Δ Discriminated between Different Conformations of the β2AR

**Figure 2:**
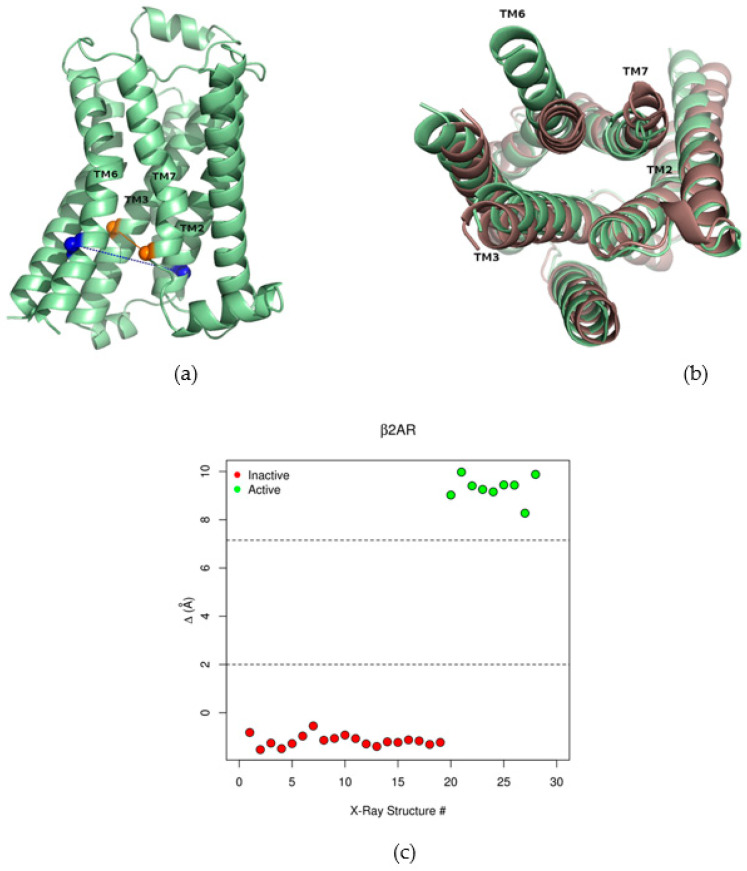
The collective variable Δ. (**a**) Active conformation of the β2AR, showing the residues involved in the definition of Δ. The Cα′s of residues Y70 and G276 are represented by blue spheres and the distance between them (d1) is indicated by blue dots. The Cα′s of residues C125 and I325 are shown as orange spheres and the distance between them (d2) is indicated by orange dots. Δ = d1 − d2; (**b**) Superposition between inactive (red) and active (green) conformations of β2AR, showing the outward movement of TM6 and the inward movements of TM7 and (to a lesser extent) TM3, that increase Δ upon activation. (**c**) Values of Δ for inactive (red) and active (green) experimental structures of β2AR. The order of structures coincides with Table S1.

To characterize the conformational state of a GPCR, a collective variable denominated Δ has been proposed by the GPCRdb webserver [27]. As explained in the Methods section, it consists in the difference between two distances: Δ = d1 − d2; d1 is the distance between transmembrane helices (TMs) 2 and 6, and d2 is the TM3-TM7 distance. In the β2AR, these two distances are measured between the Cα′s of residues Y70-G276 and C125-I325, respectively. Figure 2a shows the Cα′s of these residues in an active conformation of β2AR. The Y70-G276 pair is represented by blue spheres, while C125 and I325 are depicted in orange. The dotted lines connecting these residues highlight the distances that define Δ. This variable captures key conformational rearrangements that occur upon receptor activation: (i) the increase in the TM2-TM6 distance, due to the outward movement of TM6; (ii) the decrease in the TM3-TM7 distance, resulting from an inward movement of these helices. These conformational changes are shown in Figure 2b, that illustrates a superposition between inactive (in red) and active (in green) conformations of β2AR. By taking into account the motions of distinct TMs, the variable Δ encapsulates the collective character of the conformational transition that accompanies the receptor activation. As shown in Figure 2c, Δ effectively separates experimental structures of β2AR according to their conformational state. Active conformations are represented by green circles and have high values of Δ (greater than 8 Å, denoting large TM2-TM6 and shorter TM3-TM7 distances, typical of the active state); in contrast, inactive structures (red circles) display negative values of Δ.

### 2.2. Correlations between Δ and Inter-Residue Distances in the β2AR

**Figure 3:**
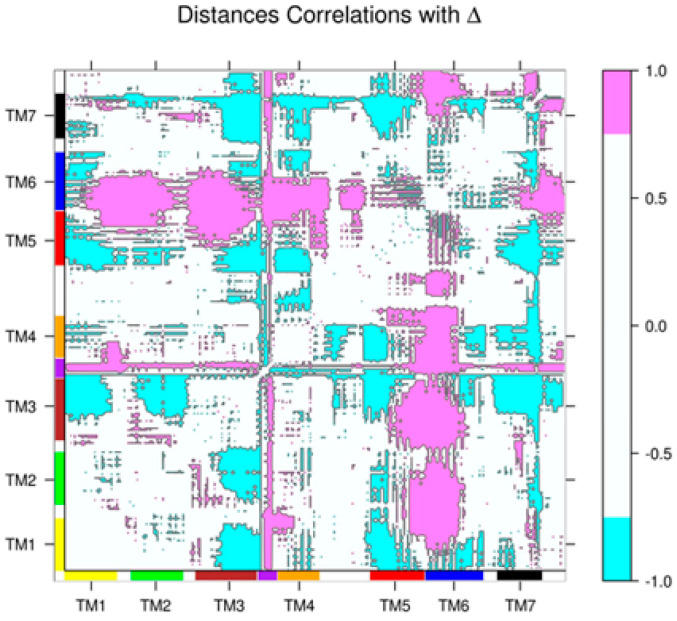
Matrix of correlations between inter-residue distances and Δ for the ensemble of experimental structures of the β2AR. Each element *cij* in this matrix is the correlation coefficient between Δ and the distance between residues *i* and *j.* Only strong correlations (|*cij*| > 0.75) are shown. Positive correlations are in pink and anti-correlations are in cyan. Segments corresponding to transmembrane helices are indicated by color rectangles on the margins of the figure (ICL2 is represented by the purple rectangle between TM3 and TM4).

To assess dynamical couplings of distinct regions of the receptor to the collective activation motion, we measured the correlation between each inter-residue distance and Δ, since, from a geometric point of view, this variable correlates with the conformational state of the receptor. These correlations were measured in the ensemble of experimental structures of β2AR, containing inactive and active conformations (see Section 4; the PDB codes of the structures in this ensemble are in Table S1), and resulted in a matrix where each element *cij* was the correlation coefficient between Δ and the distance between residues *i* and *j*. This matrix is available as Supplementary Data and is illustrated in Figure 3, where strong correlations with Δ (*cij* > 0.75) are depicted in pink and strong anti-correlations with Δ (*cij* < −0.75) appear in cyan. Positive correlations indicate distances that increase upon receptor activation, while the opposite is true in the case of anti-correlations. It is interesting to note that distinctive features of β2AR activation can be observed in the matrix: (i) the increased distance between TM6 and TM1 to TM4, indicative of the outward motion of TM6; (ii) the inward motions of TM3 and TM7; (iii) increased distances between parts of the intracellular loop 2 (ICL2, situated between TM3 and TM4 and indicated by purple rectangles in the margins of the figure) and the rest of the structure, due to an important rearrangement of this loop: it goes from a disordered to a helical conformation when β2AR is activated (compare Figure 1b,c). The strong correlations involving this loop proved important in the identification of an allosteric site in this region, as shown below.

### 2.3. Correlations with Δ Allowed the Identification of Allosteric Sites in the β2AR

**Figure 4:**
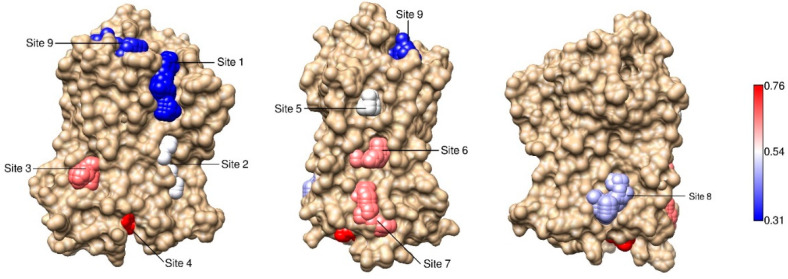
β2AR sites couplings to Δ. Sites on the surface of β2AR are represented by spheres, that are colored according to C_site_, the coupling strength (from blue, denoting weaker coupling, to red, indicating stronger coupling). Sites 4, 6 and 7 correspond to known allosteric sites (see Figure 1a–c).

In order to predict potential allosteric sites, we focused our attention on inter-residue distance fluctuations within cavities of the β2AR. We restricted our analysis to non-orthosteric sites on the surface of the receptor, including the intracellular and extracellular regions and the interfaces between the TMs and the membrane. First, we identified these sites using Mdpocket [28], a software that determines cavities in ensembles of structures. We found nine pockets across the receptor structure (Figure 4), that were labeled as Sites 1–9. The residues belonging to each site are listed in Table S2.

Notably, Sites 4, 6 and 7 corresponded to the locations of known allosteric sites (Figure 1a–c). Site 4 was located on the intracellular side, overlapping with the G Protein binding site and also with the region that interacts with the allosteric ligand in PDB structure 5X7D [22]. Site 6 was situated between TM4 and TM5, where the allosteric modulator in PDB structure 6OBA is bound [24]. Site 7 corresponded to the allosteric site in PDB 6N48 [23], also between TM4 and TM5 and close to ICL2.

To estimate the coupling of a given site to the collective conformational change associated with the activation of the receptor, we extracted from the matrix described in the previous section only those elements corresponding to residues of the site. Therefore, for each site we built a sub-matrix whose elements *cij* were correlations between Δ and the distance between residues *i* and *j* within the site. Then, we averaged the absolute value of the correlations in the sub-matrix, to obtain a single number that conveniently summarized the coupling strength of the site. This average coupling was denominated C_site_ (see Equation (3)), and was used to rank the sites, as shown in Table 1 and illustrated in Figure 4, where the spheres representing the sites are colored according to the coupling strength (blue represents weaker coupling, white indicates intermediate values and red denotes stronger coupling).

In Table 1, the mean value of C_site_ is 0.51. Interestingly, Sites 4, 6 and 7, that corresponded to known allosteric sites, were found on the top of the ranking, with C_site_ values above the mean. This result suggests that allosteric sites in β2AR can be predicted based on dynamical correlations and that sites with stronger couplings can be regarded as potentially allosteric.

**Figure 5:**
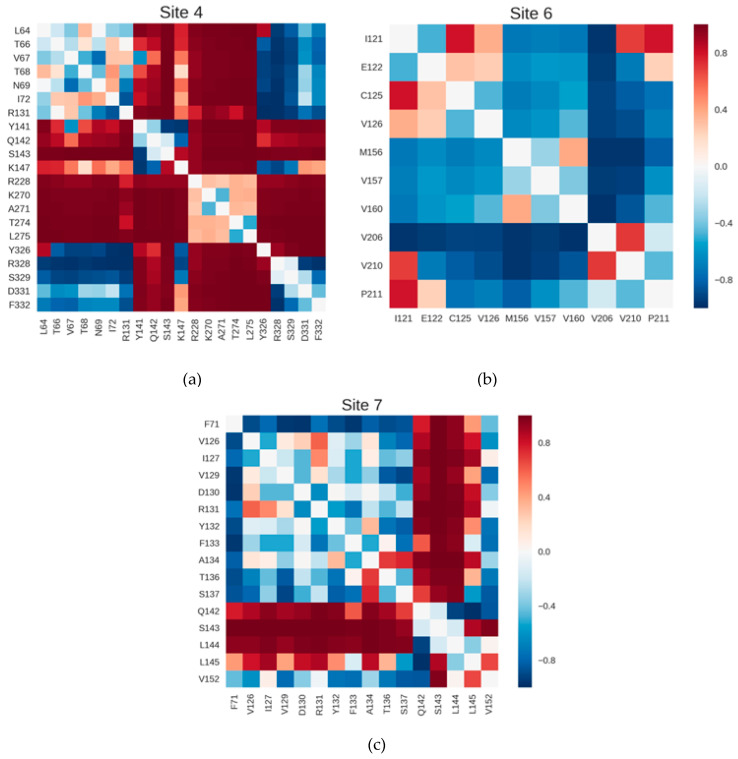
Matrices of correlations between inter-residue distances and Δ for top-ranked sites in the β2AR. (**a**) Site 4; (**b**) Site 6; (**c**) Site 7. These sites show strong coupling to Δ and correspond to known allosteric sites. Each element *cij* in these matrices is the correlation coefficient between Δ and the distance between residues *i* and *j.* Strong anti-correlations are in blue, intermediate values in white and strong correlations in red.

A closer look into the correlation sub-matrices of each site permitted a more detailed analysis of the dynamical profile of the pockets. As an illustration of this analysis, we focused on Sites, 4, 6 and 7, that showed values of C_site_ above the mean in Table 1 and corresponded to known allosteric sites. Figure 5a–c depicts the sub-matrices of these sites; those of the remaining sites are shown in Figure S2. The color scale is the same in all these figures, with strong anti-correlations with Δ in blue, intermediate values in white and strong correlations in red. In general, the darker shades in the matrices of top-ranked sites show that they tend to display not only individual distances with stronger couplings, but also many pairwise distances contributing to the overall coupling coefficient. Particularly in Sites 4, 6 and 7 (Figure 5a–c), it is possible to see groups of residues whose distances to most other residues in the site are strongly coupled to Δ. This observation suggests a higher collectivity in the dynamical behavior of these sites in comparison to weaker pockets.

**Figure 6:**
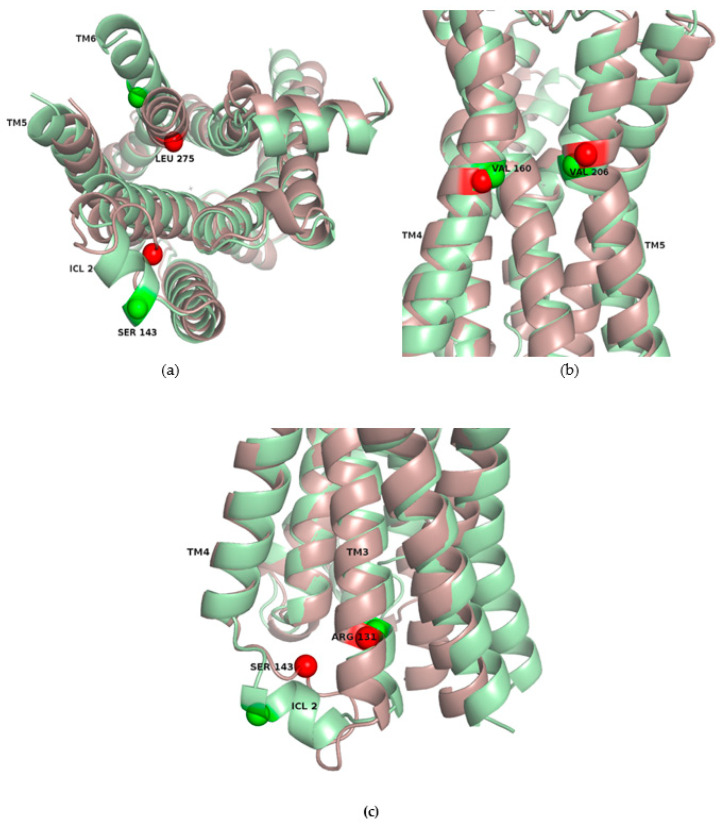
Pairwise distances highly correlated to Δ in β2AR top-ranked sites. (**a**) L275 and S143, in Site 4; (**b**) V160 and V206, in Site 6; (**c**) S143 and R131, in Site 7. The distances between these residues contribute the most to the coupling strength of the respective sites. The Cα′s of these residues are shown as spheres, and inactive (red) and active (green) conformations are superposed, to highlight the conformational changes of the sites upon receptor activation.

The inspection of the sub-matrices also revealed the pairwise distance that contributed the most to the allosteric coupling in each site and facilitated the analysis of the local conformational changes that are coupled to the global activation transition. For Sites 4, 6 and 7, the residue pairs involved in such distances are shown in Figure 6a–c, where the inactive (red) and active (green) conformations are superposed, and the Cα’s of the relevant residues are depicted as spheres. In Site 4, the distance between L275, in TM6, and S143, in ICL2, presented the highest correlation with Δ (Pearson correlation coefficient *R* = 0.99). The increase in this distance upon receptor activation is due to the outward movement of TM6 and also to the conformational rearrangement of ICL2, that adopts a helical structure (Figure 6a). If this site is occupied by an allosteric ligand when the receptor is inactive, as in PDB 5X7D, these conformational changes linked to the activation motion are potentially blocked, besides the steric hindrance to G Protein binding. In the case of Site 6, the distance between V160 (TM4) and V206 (TM5) was strongly anti-correlated with Δ (*R* = −0.97), indicating a constriction of the site when the receptor is activated (Figure 6b). The binding of a modulator to the inactive conformation of the pocket (as observed in PDB 6OBA) might inhibit this relative movement of TM4 and TM5 that occurs in activation. In Site 7, as already remarked above, the rearrangement of ICL2 was crucial in providing this region with its allosteric role. Accompanying the folding of the loop into a helix, S143 moves away from R131 (in TM3) upon β2AR activation; this distance was strongly correlated with Δ (*R* = 0.99), indicating that the relative movement of these residues occurs in concert with the activation. Blocking of these local motions by an allosteric modulator (such as the one bound to an active conformation of β2AR in PDB 6N48) could interfere with the global dynamics of the receptor. Therefore, the analysis of these three sites suggests that certain conformational states of the receptor can be stabilized by preventing or blocking local motions.

### 2.4. Allosteric Sites in the β2AR Could Be Detected by Normal Mode Analysis

The relative abundance of structural information on the β2AR, with several inactive and active X-ray structures of the receptor, is not available for most GPCRs. In many cases, only inactive conformations have been obtained experimentally. Therefore, to evaluate the possibility of predicting allosteric sites in other receptors, we investigated if NMA could capture the dynamical couplings observed in the experimental ensemble of β2AR. NMA is a useful method to study collective dynamics and allosteric couplings in proteins, including GPCRs [29,30]. We calculated the normal modes of the inactive conformation and generated a harmonic trajectory that was analyzed following the same procedures described above. The results, shown in Table S3, revealed a poor agreement with the experimental ensemble. From the known allosteric sites, only Site 4 showed C_site_ above the mean value of the ranking (which coincided with C_site_ of Site 6). Moreover, the values of the couplings across the table were low.

Since the vibrational analysis can provide a local conformational exploration around each structure, we hypothesized that a combination of conformations obtained from NMA of the inactive and active states could approximate the heterogeneity of the experimental ensemble of the β2AR, where the prediction of allosteric sites was successful. Then, we concatenated harmonic trajectories from the inactive and active conformations, which led to better results, with stronger couplings and Sites 4 and 7 on the top of the ranking (Table S4). The values of Δ in these harmonic ensembles can be seen in Figure S3. However, the importance of Site 6 was underestimated in these calculations. Despite this limitation, a combined examination of these results and of FTMap and FTSite predictions [14,15,16] allowed the correct identification of the known allosteric sites. We used as inputs for these programs the same structures employed for NMA (PDBs 2RH1 [31] and 4LDO [32] for inactive and active conformations, respectively). Site 6 was highly ranked by FTMap and FTSite in the active structure; on the other hand, both programs underestimated the role of Site 7 in comparison to our approach (Figure S4). Therefore, Sites 4, 6 and 7 could all be found among top ranked sites either in dynamical coupling or FTMap/FTSite analyses.

To verify if our calculations provided meaningful and complementary information with respect to other established computational tools, we also compared our results to predictions from PARS [17] and SPACER [18]. The inputs for all these programs were the same as above (PDBs 2RH1 and 4LDO, for inactive and active conformations, respectively). As shown in Figures S5 and S6, PARS and SPACER did not capture the importance of Sites 6 and 7. In addition, we also compared our predictions to those of D3Pockets [21], that calculates correlations between volumes of binding sites. The input in this case was the ensemble of experimental structures of the β2AR. The correlations measured by D3Pockets did not point directly to the known allosteric sites or their involvement in the collective motion of activation (Figure S7).

We also analyzed ensembles generated by MD simulations. We ran short (200 ns) trajectories of the inactive and active conformations of the β2AR. Three independent simulations were performed for each conformational state, and the trajectories were combined in nine distinct ensembles, containing inactive and active conformations. The time evolution of RMSD and Δ in these trajectories can be found in Figure S8. Analysis of dynamical couplings in the nine ensembles showed that Sites 4 and 7 were highly ranked, but Site 6 presented a relatively low value of C_site_ (Table S5). Overall, MD simulations did not improve the results obtained by NMA, and the harmonic analysis provided a better agreement with the couplings obtained in the experimental set. This suggests an insufficient sampling in MD simulations, that would require longer trajectories to adequately capture the dynamical correlations.

### 2.5. The Known Allosteric Site of GCGR Was Identified by Dynamical Correlations

The results obtained with NMA of the β2AR encouraged the application of the approach to other GPCRs, even when few experimental structures are available. Then, to further verify the usefulness of dynamical correlations in identifying allosteric sites, we have chosen the glucagon receptor (GCGR), a class B GPCR. The allosteric site in this receptor was not detected by FTMap [13], since this program was not specifically parameterized to predict binding sites in hydrophobic regions such as the interface between protein and membrane where allosteric ligands bind GCGR (Figure 1d).

For class B GPCRs, the collective variable Δ is also defined as the difference between TM2-TM6 and TM3-TM7 distances (see the Methods section for the slight differences to class A receptors). Then, it was possible to follow the procedure described for the β2AR. First, we performed NMA of GCGR in the inactive and active conformations and generated an ensemble by concatenating both harmonic trajectories. Next, we identified binding pockets across this ensemble and calculated the correlations of Δ with pairwise distances within the pockets. The residues in each identified site can be found in Table S6; the variation of Δ in the harmonic ensemble can be seen in Figure S9, and the correlations of Δ with inter-residue distances are available as Supplementary Data.

**Figure 7:**
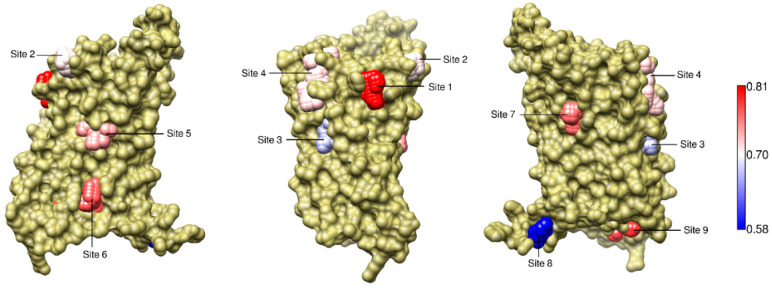
GCGR sites couplings to Δ. Sites on the surface of GCGR are represented by spheres, that are colored according to C_site_, the coupling strength (from blue, denoting weaker coupling, to red, indicating stronger coupling). Sites 5 and 6 correspond to known allosteric sites (see Figure 1d).

**Figure 8:**
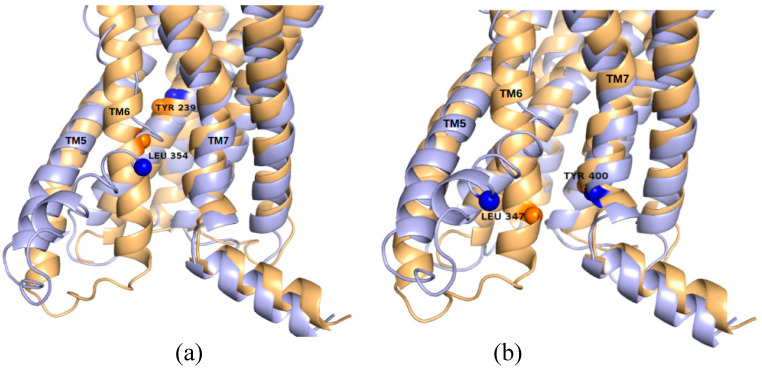
Pairwise distances highly correlated to Δ in GCGR top-ranked sites. (**a**) Y239 and L354, in Site 5; (**b**) L347 and Y400, in Site 6. The distances between these residues contribute the most to the coupling strength of the respective sites. The Cα′s of these residues are shown as spheres, and inactive (light orange) and active (blue) conformations are superposed, to highlight the conformational changes of the sites upon receptor activation.

Here, we restricted our search to the transmembrane domain of GCGR and identified nine non-orthosteric sites on its surface. Their couplings to Δ are shown in Table 2. The pocket labeled as Site 6 occupied the third position of the ranking, with C_site_ above the mean value (0.73) in the table; notably, as can be seen in Figure 7, this site corresponded to regions in contact with allosteric ligands observed in X-ray structures (see Figure 1d). Interestingly, NMA of the inactive conformation also revealed a strong coupling of Site 6, which occupied the top of the ranking (Table S7). Moreover, the second place in this ranking was occupied by Site 5, also situated between TM6 and TM7 and in contact with known allosteric ligands. Site 5 also showed C_site_ above the average value of the ranking in the combined harmonic trajectory (Table 2). Sites 5 and 6 were found in a region that suffers important conformational changes during receptor activation. This can be seen in Figure 8, that illustrates a superposition of inactive and active conformations and shows, for each of the two sites, the distance with the highest correlation with Δ. In Site 5, the distance between Y239 (in TM3) and L354 (in TM6) was strongly correlated with Δ (*R* = 0.99); the same occurred for the distance between L347 (in TM6) and Y400 (in TM7) in Site 6 (*R* = 0.99). Occupation of these sites can block the outward movement of TM6 and stabilize the receptor in an inactive conformation, as observed in PDBs 5EE7 and 5XEZ [25,26].

Following the same procedure adopted for the β2AR, we analyzed dynamical couplings in ensembles generated by MD simulations. Site 6 was the second in this ranking (Table S8), that showed overall results comparable to those obtained by NMA. However, we note that the short trajectories (200 ns), particularly those of the active conformation, suffered from poor equilibration (Figure S10), indicating the necessity of longer time scales for more robust conclusions.

### 2.6. Dynamical Correlations Identified a Known Allosteric Site in NMA of the M2 Muscarinic Receptor

As a final example, we performed NMA of the M2 muscarinic receptor, following the same protocol adopted for β2AR and GCGR: we concatenated harmonic trajectories from the inactive and active conformations and identified cavities on the surface of the receptor (the values of Δ in this ensemble are shown in Figure S11). From the nine cavities identified, the one labeled as Site 1 overlapped with an allosteric site observed in the active state of M2, in PDB structure 4MQT [33] (Figures S1 and S12; Table S9 enumerates the residues in the sites). Table S10 shows that Site 1 was the second in the ranking, with C_site_ = 0.72, above the mean value in the ranking (0.64). Therefore, it was correctly identified as potentially allosteric. However, contrary to GCGR, NMA of the inactive conformation failed to identify this allosteric site: it was only the fifth in the ranking, with the C_site_ below the mean (see Table S11). In contrast to allosteric sites in β2AR and GCGR, this site is not in the intracellular or transmembrane domains, but it is located in a vestibule just above the orthosteric site. The well described contraction of this vestibule in the active state [28] was only captured by our analysis when we combined the harmonic trajectories from both conformational states, and also in an ensemble of experimental structures (see Table S12 for the included PDBs and Table S13 for the ranking of sites). Dynamical couplings in ensembles generated by MD simulations failed to detect the allosteric site, even with the mixed set of inactive and active conformations (Figure S13 and Table S14). This difficulty is further discussed below.

## 3. Discussion

In this work we identified potential allosteric sites in GPCRs by evaluating the coupling of internal fluctuations of pockets to the global dynamics of the receptor. We calculated correlations between inter-residue distances and a collective variable, denominated Δ, describing the conformational state of the receptor. Our main case study was the β2AR, a model class A GPCR. Currently, there are 34 structures of the β2AR available in the PDB, three of them in the presence of allosteric modulators. This relatively abundant structural information allowed us a comparison between computational calculations and experimental data. We also studied the GCGR, a class B GPCR presenting an allosteric site whose prediction failed in an earlier analysis based on druggability estimations [13], and the M2 muscarinic receptor. For these receptors, we successfully identified the known allosteric sites among the top sites in a ranking measuring the strength of coupling to the collective variable Δ and, therefore, to the global activation motion.

Our approach was based on a fundamental link between long-range correlations and allostery, a subject that has been extensively investigated in the literature [34,35]. In particular, analyses of dynamical correlations between distant regions or modifications of protein dynamics through perturbations of specific sites are at the heart of recent computational methods that aim at the identification of allosteric sites [36,37,38,39]. We share with such methods the appreciation of protein dynamics as one of the fundamental ingredients of allosteric modulation. However, in contrast to them, our approach was thought specifically for GPCRs and explored hallmarks of the activation dynamics of these receptors. Nevertheless, we believe the same fundamental ideas employed here could be applied to other proteins as well, provided a convenient collective variable (analogous to Δ) could be found.

One of the fundamental steps of our calculations was the identification of sites on the surface of the receptors. We used the software Mdpocket [28] to detect common sites in different conformations of the receptors, but other tools could be used, and other types of sites could be looked for (e.g., deep or cryptic sites). Difficulties in the delimitation of pockets, resulting in excessively large sites, affected the results we observed with D3Pockets [21] and can help to explain why this program did not clearly show dynamical couplings relevant to the activation motion.

The rankings of the sites, that we used as a reference, should not be seen as an absolute classification or a rigid separation between allosteric and non-allosteric sites, but rather as a relative scale indicating the likelihood that a given site interferes with the dynamics of the receptor. In this perspective, the division between C_site_ values above and below the mean in each ranking (Table 1 and Table 2) showed predictive value, since known allosteric sites were found among those with couplings stronger than the average. However, an allosteric role played by a low-ranked site cannot be discarded, as demonstrated by our results obtained for Site 6 in NMA or MD simulations of the β2AR.

In general, our predictions were better when we analyzed ensembles containing both inactive and active conformations. Except for GCGR, the analysis of dynamical couplings in NMA of inactive conformations resulted in poor predictions of the allosteric sites. This indicates a limited value of our approach in cases where only inactive structures are available. In the case of the M2 receptor, the results from NMA of the inactive conformation and from mixed ensembles generated by MD simulations deserve to be considered in more detail, as they draw attention to another possible limitation of the method discussed in this paper. Contrary to what is observed in the β2AR or the GCGR, the allosteric site of M2 is situated in the entry of the orthosteric site, in the extracellular side of the receptor. The comparison between inactive and active conformations of M2 shows that this allosteric pocket is pre-formed when an agonist is bound to the orthosteric site [33]; the allosteric modulator further stabilizes the active, closed conformation of this vestibule and of the orthosteric site. The closure of the vestibule is mainly due to inward motions of TM6 and extracellular loop (ECL) 3. Our observations of NMA of the inactive conformation and of MD simulations of M2 point to the confirmation of a previously formulated hypothesis [33], namely, a flexibility of TM6 leading to independent conformational changes in the region of the orthosteric site and in distal regions of the receptor. This ultimately led to a weak coupling between the internal fluctuations of the allosteric site and the collective variable Δ. This result is a warning that our approach may be more indicated to the detection of allosteric sites in the transmembrane or intracellular domains, in contrast to those adjacent to the orthosteric site. Indeed, the coupling between the extracellular domain and the G protein binding site has already been characterized as loose [40,41], and therefore it should be much harder to capture by our approach, that is entirely based on the strength of dynamical correlations. In such situations, tools that estimate druggabilities can be advantageous; FTMap, for example, correctly identified the allosteric site of the M2 receptor in a previous study [13].

In the case of the β2AR, NMA of the inactive state failed to predict Site 7. This site undergoes an important rearrangement upon activation, as ICL2 goes from a disordered conformation to a helix. This transition underlies the high C_site_ value observed for this site in the set of experimental structures but is very challenging to predict from the simulations of the inactive structure alone. The role of this site in the collective dynamics of the receptor could only be captured when we considered ensembles containing inactive and active structures. Site 6, in contrast, was not highlighted in neither of the cases. In the case of NMA, this could result from shortcomings of the harmonic approximation: NMA is a useful tool to study large amplitude collective motions in proteins, and functional transitions are often described by a few low-frequency modes [29]. However, localized, high-frequency or anharmonic fluctuations can also be functionally relevant to GPCRs. Such fluctuations can occur in binding sites and may not be adequately captured by the harmonic approximation. This points to the important topic of the coupling between local and global fluctuations, a subject extensively studied in different contexts [42,43]. In the case of GPCRs, we believe it deserves further investigation.

In principle, MD simulations would be a good choice to overcome such limitations of the harmonic approximation. However, our results demonstrated that the time scales simulated here (200 ns) may not be sufficient for proper equilibration and sampling of dynamical correlations in the receptors we investigated. Ideally, the trajectories should be extended, but this can represent an important increase in computational cost. NMA was a reasonable compromise, keeping the protocol relatively inexpensive.

Despite these limitations, we succeeded in identifying the allosteric site of GCGR, that FTMap failed to predict. Moreover, in the β2AR our approach clearly indicated the relevance of the allosteric sites identified here as Sites 4 and 7; and combining our results with those provided by FTMap permitted to detect Site 6. On the other hand, FTMap classified Site 7 as weak (ranked 10 in a list of 14). Therefore, when taken together, these results point to the relevant complementary role played by the method presented here. Our approach has also provided better results than PARS and SPACER, that missed both sites 6 and 7. This limitation can possibly be related to the fact that these methods evaluate different dynamical features and were not designed to investigate GPCRs. It should also be considered that both of these methods rely on coarse-grained normal mode analysis to account for protein motions, in contrast to our more detailed all-atom, force field based NMA calculations. However, a systematic comparison between coarse-grained and all-atom NMA to describe GPCR dynamics is beyond the scope of this paper.

The crystal structures of the β2AR obtained in the presence of allosteric modulators demonstrate the existence of three distinct allosteric sites in the same receptor. Moreover, these sites are situated in the transmembrane and intracellular domains, indicating that not only extracellular sites should be considered when searching for allosteric ligands. In agreement with this observation, our calculations point to the existence of multiple allosteric sites in GPCRs, in different regions of the receptor structure. This multiplicity of potential allosteric sites may represent more possibilities of therapeutic intervention; it could also suggest complex scenarios where more than one modulator simultaneously binds to a same GPCR. The receptor’s response would depend on the nature of the modulators, of the orthosteric ligand and the membrane environment since, as previously remarked, a receptor-ligand(s) complex is essentially a new system, with unique pharmacological properties [44]. This delicate interplay between the distinct components of the system can be illustrated by the phenomenon of probe dependence: the effect of a given allosteric modulator depends on the orthosteric ligand used to probe the receptor′s function [45]. Extending the same idea to a situation where more than one allosteric modulator is present, the outcome would depend on the particular combination of ligands bound to the GPCR.

The occurrence of several allosteric sites also raises the question of their possible regulation by endogenous ligands. Although allosteric sites in GPCRs did not necessarily evolve to host endogenous modulators, it is possible that endogenous molecules might play a role in allosteric GPCR regulation, as occurs in other receptor superfamilies (e.g., nuclear hormone receptors) [46]. Indeed, there is growing evidence for the involvement of proteins, peptides, amino acids, ions and lipids in the allosteric modulation of GPCRs [46]. In addition to the extracellular domain, this diverse array of molecules can also interact with transmembrane and intracellular sites.

Besides G proteins and β-arrestins, which are striking examples of endogenous allosteric modulators, other accessory proteins, such as RAMPs (receptor activity-modifying proteins) and melanocortin receptor accessory proteins (MRAPs) can also modulate the signaling of GPCRs [46]. Among the ions, it is well known that sodium can act as a negative allosteric modulator of several class A GPCRs, and a conserved binding for Na+ has been identified in X-ray crystal structures (e.g., in β1AR and δ-opioid receptor [47,48]). In the case of β2AR, it has been shown that zinc can also modulate the receptor, regulating orthosteric ligand binding or the effect of the agonist [49,50]. The role of lipids in GPCR function has been increasingly recognized, and simulations show their influence in receptor′s dynamics and activation [51,52,53,54,55]. In particular, cholesterol can not only modify physical properties of the membrane, but also directly bind to the receptor. In a crystal structure of β2AR, two molecules of cholesterol interact with a region comprising residues from TMs 2 and 4, containing a putative cholesterol consensus motif (CCM) [56]. Other lipids, such as endocannabinoids, have also been shown to modulate ligand binding in noncannabinoid GPCRs [43].

Importantly, molecules that are generated in pathological conditions, such as inflammatory peptides or GPCR-targeted autoantibodies, may also act as endogenous modulators [46].

Therefore, the existence of multiple allosteric sites in a same receptor raises the possibility that the effect of an allosteric drug depends on other endogenous allosteric molecules. Computational methods can be useful to investigate how the mutual interactions between allosteric modulators affect GPCR dynamics [57]. The dynamical integration of these different signals can lead to complex cellular responses, that depend on the spatiotemporal combination of exogenous and endogenous factors interacting with the receptor.

Finally, we should acknowledge the remarkable complexity of allostery. Fundamentally, allosteric modulation results from a modification of the underlying free energy surface of the system. The structure as well as the dynamical behavior of the protein can change as a consequence of the modified energetics [58]. Different mechanisms of allosteric modulation can underlie the observed effect, that emerges from an intricate interplay between the protein, ligands, other proteins, solvent and, in the case of GPCRs, the membrane. In this context, correlated motions in the protein can be one manifestation of the phenomenon. The method outlined in this paper can be a useful auxiliary tool to further explore this particular aspect, and it might contribute to better understand allostery in GPCRs.

## 4. Materials and Methods

### 4.1. Structural Ensembles

#### 4.1.1. Experimental Ensemble of the β2AR

The analyzed ensemble was composed of 28 experimentally determined structures of the β2AR, including inactive and active conformations. Structures containing allosteric modulators (5X7D, 6N48 and 6OBA) as well as structures missing several residues around the orthosteric site and the extracellular loops (2R4R, 2R4S, 3KJ6) were not included. The PDB codes of the structures retained for analysis are listed in Table S1. Modeller [59] was used to revert mutated residues to their wild type counterparts and add missing residues. The analyzed structures contained residues 32–227 and 267–342 (the ICL3 was omitted).

#### 4.1.2. Experimental Ensemble of the M2 Muscarinic Receptor

It was composed of 10 experimental structures of M2, including inactive and active conformations. The PDB codes of the structures retained for analysis are listed in Table S12. Modeller [59] was used to add missing residues. The analyzed structures contained residues 23–213 and 386–456 (the ICL3 was omitted).

### 4.2. Normal Mode Analysis

All-atom NMA of all receptors was performed with the CHARMM program [60]. Calculations were performed on models based on the inactive and active conformations of each receptor. For the β2AR, PDB files 2RH1 (inactive) and 4LDO (active) were used as inputs. For GCGR, PDB files 5XF1 [26] and 6LMK [61] were the models for the inactive and active conformations, respectively. For M2, models based on PDB files 3UON (inactive) [62] and 4MQS (active) were used [33]. Receptors were subjected to NMA in their apo form, and any bound ligands or proteins were removed.

For β2AR and M2, ICL3 was replaced by a stretch of six alanine residues connecting TM5 and TM6, as previously described [63].

In all cases, the protein was described by the CHARMM36 force field [64,65]. Initially, the structure was energy minimized in vacuo with the Adopted Basis Newton–Raphson algorithm until an energy gradient of 10^−6^ kcal.mol^−1^.Å^−1^ was reached. Diagonalization of the Hessian matrix was performed with the command *diag*, implemented in CHARMM. A harmonic trajectory was generated by the linear combination of the 100 lowest frequency normal modes, using the *write trajectory* command implemented in the *vibran* module of CHARMM. We then concatenated the harmonic trajectories generated for the inactive and active conformations, and this concatenated trajectory was analyzed.

### 4.3. Molecular Dynamics Simulations

For each receptor, and for each conformational state, we ran three independent replicas of 200 ns. After that, these trajectories were combined in nine distinct conformational ensembles, containing a mixed set of inactive and active conformations. The analysis of dynamical couplings was performed in these mixed ensembles.

The inputs for MD simulations were the same PDB structures used for NMA (these structures are depicted in Figure S14). To further stabilize the conformations, the inactive and active structures of β2AR and M2 were simulated with the ligands bound to the orthosteric sites of the respective PDB structures. Moreover, the active conformations of these receptors were simulated in the presence of a nanobody coupled to the receptor in the original PDBs. The active conformation of GCGR was simulated in the presence of glucagon bound to the orthosteric site. Table S15 summarizes the setup of all simulations. For each system, a representative snapshot (the final frame of one of the replicas) is available as a supplementary file.

All MD simulations followed the same protocol. Initially, the receptor model was aligned according to the Orientations of Proteins in Membranes database [66]. Then, the system was prepared with the HTMD software [67]. Ligands were removed and the apo receptor was inserted in a pre-equilibrated 90 × 90 Å^2^ POPC membrane, solvated with TIP3P water molecules [68] and ionized up to a concentration of 0.15 M NaCl. Protonation states were determined by Propka [69,70] at pH 7.0 and chain termini were capped with neutral groups. Proteins, lipids and ions were described by the CHARMM36 force field [64,65]. Simulations were performed with the ACEMD3 software [71]. After 500 steps of energy minimization, 40 ns of equilibration in the NPT ensemble were performed. Temperature was kept at 300 K by a Langevin thermostat and pressure was maintained at 1.0 bar by a Monte Carlo barostat. Initially, the Cα′s were restrained by a 1.0 kcal.mol^−1^. Å^−2^ force constant, and the other heavy atoms were restrained by a 0.1 kcal.mol^−1^. Å^−2^ force constant. These restraints were progressively decreased to 0 until 20 ns; after that, equilibration proceeded unrestrained from 20 ns to 40 ns. Following equilibration, a 200 ns production run was performed in the NVT ensemble (T = 300 K). Only the last 100 ns were retained for analysis. In all stages, hydrogen mass repartitioning and a time step of 4 fs were employed, and snapshots were saved every 100 ps. A 9 Å cut off was used for non-bonded interactions and PME (Particle Mesh Ewald) [72] was used for long-range electrostatic interactions.

### 4.4. The Collective Variable Δ

This variable distinguishes, from a purely geometric point of view, between inactive, intermediate and active conformations of the receptor and is defined as:

Δ = d1 *−* d2, (1)

where d1 is a distance between the Cα′s of residues in TM2 and TM6 and d2 is a distance between the Cα′s of residues in TM3 and TM7. For class A GPCRs, d1 is measured between residues 2 × 41 and 6 × 38 and d2 is the distance between residues 3 × 44 and 7 × 52. For class B receptors, d1 is measured between 2 × 41 and 6 × 33, while d2 is established between 3 × 44 and 7 × 51. These residue numbers correspond to the GPCRdb numbering scheme [27,73].

Table 3 below summarizes the residues used to determine Δ in the receptors studied in this work.

### 4.5. Calculation of Inter-Residue Distances

Euclidean distances were measured between the residues’ Cα’s. For every structure, a distance matrix was obtained through the *dm* command of the Bio3D software [74]. Therefore, each analyzed ensemble was represented by a set of distance matrices, one matrix for each structure in the ensemble. Distances between a given pair of residues across an ensemble were extracted from the distance matrices.

### 4.6. Coupling between Inter-Residue Distances and Δ

The coupling between a given distance and the variable **Δ** was determined by the Pearson correlation coefficient, using the *cor* command implemented in R. Therefore, for a distance *dij* between residues *i* and *j* the coupling to Δ was given by

(2) $$C_{ij}=\frac{cov\left(d_{ij},\Delta \right)}{\sigma_{dij}\sigma_{\Delta }},$$

where *cov(dij,*Δ) is the covariance and *σ_dij_* and *σ*_Δ_ are the standard deviations of *dij* and Δ, respectively.

### 4.7. Determination of Receptors′ Sites

Prior to site detection, each ensemble was aligned based on the Cα′s of the receptor. Sites were detected in each ensemble with the Mdpocket software [28]. Only sites that were present in at least 40% of the structures in a given ensemble were retained. Moreover, only sites exposed on the surface of a receptor were considered for analysis, while deep, buried sites were discarded. For the β2AR, sites were detected in the ensemble of experimental structures. For GCGR and the M2 muscarinic receptor, sites were determined in the respective concatenated NMA trajectories containing both inactive and active conformations.

A residue was considered part of a site if any of its atoms were within 4.0 Å of any of the dummy atoms with which Mdpocket fills the site.

### 4.8. Coupling between Sites and Δ

To obtain the coupling profile of one site, we summed the absolute values of couplings of the pairwise distances within the site and divided the sum by the number of residue pairs in the site:

(3) $$\begin{array}{c} C_{\mathrm{site}}\;=\;\frac{2}{N\left(N-1\right)}\underset{i,j>i}{\sum }\left|C_{ij}\right| \end{array}\;,$$

where *N* is the number of residues in the site and *Cij* is the correlation between Δ and the distance between residues *i* and *j* of the site.

## Figures and Tables

**Table 1:** Ranking of β2AR sites based on the strength of their coupling to Δ.

Site Label	Coupling with Δ (C_site_)
4 *	**0.76**
**3**	**0.61**
7 *	**0.60**
6 *	**0.59**
5	0.50
2	0.49
8	0.44
9	0.33
1	0.31

Sites with C_site_ above the mean of the ranking are highlighted in bold. Those marked with * overlap with known allosteric sites.

**Table 2:** Ranking of GCGR sites based on the strength of their coupling to Δ in normal mode analysis (NMA).

Site Label	Coupling with Δ (C_site_)
**1**	**0.81**
**9**	**0.79**
6 *	**0.77**
**7**	**0.76**
5 *	**0.74**
4	0.72
2	0.71
3	0.67
1	0.58

Sites with C_site_ above the mean of the ranking are highlighted in bold. Those marked with * overlap with the known allosteric site.

**Table 3:** Residues that define Δ.

Receptor	TM2	TM6	TM3	TM7
β2AR	Y70	G276	C125	I325
GCGR	A175	L347	L243	V398
M2	Y60	L390	L115	C439

## Data Availability

Data presented in this study are available as supplementary material.

## Supplementary Materials

Supplementary materials can be found at https://www.mdpi.com/1422-0067/22/1/187/s1. Figure S1: M2 muscarinic receptor bound to agonist and allosteric modulator; Table S1: PDB codes of structures in the experimental ensemble of β2AR; Table S2: Residues in β2AR sites; Figure S2: Matrices of correlations between inter-residue distances and Δ for sites in the β2AR; Table S3: Ranking of β2AR sites based on the strength of their coupling to Δ, obtained from NMA of the inactive conformation; Table S4: Ranking of β2AR sites based on the strength of their coupling to Δ, obtained from the concatenation of NMA trajectories of the inactive and active conformations; Figure S3: Variation of Δ in NMA trajectories of the β2AR; Figure S4: FTMap and FTSite results for the β2AR; Figure S5: PARS results for the β2AR; Figure S6: SPACER results for the β2AR; Figure S7: D3Pockets results for β2AR; Figure S8: Time evolution of RMSD and Δ in MD simulations of the β2AR ; Table S5: Ranking of β2AR sites based on the strength of their coupling to Δ, obtained from MD simulations ; Table S6: Residues in GCGR sites; Figure S9: Variation of Δ in NMA trajectories of GCGR; Table S7: Ranking of GCGR sites based on the strength of their coupling to Δ, obtained from NMA of the inactive conformation; Table S8: Ranking of GCGR sites based on the strength of their coupling to Δ, obtained from MD simulations; Figure S10: Time evolution of RMSD and Δ in MD simulations of the GCGR; Figure S11: Variation of Δ in NMA trajectories of M2; Table S9: Residues in M2 sites; Figure S12: M2 sites couplings to Δ; Table S10: Ranking of M2 sites based on the strength of their coupling to Δ, obtained from the concatenation of NMA trajectories of the inactive and active conformations; Table S11: Ranking of M2 sites based on the strength of their coupling to Δ, obtained from NMA of the inactive conformation; Table S12: PDB codes of structures in the experimental ensemble of the M2 muscarinic receptor; Table S13: Ranking of M2 sites based on the strength of their coupling to Δ, obtained from the ensemble of experimental structures; Table S14: Ranking of M2 sites based on the strength of their coupling to Δ, obtained from MD simulations; Figure S13: Time evolution of RMSD and Δ in MD simulations of M2; Table S15: Summary of MD simulations; Figure S14: Input structures for MD and NMA calculations; Supplementary Data 1: Matrix of correlations between inter-residue distances and Δ for the β2AR; Supplementary Data 2: Matrix of correlations between inter-residue distances and Δ for the GCGR; Supplementary Data 3: Final structures of MD trajectories. References [31,56,75,76,77,78,79,80,81,82,83,84,85,86,87,88,89,90] are cited in Supplementary Materials.
