# Supplementary material for: Dynamical Correlations Reveal Allosteric Sites in G Protein-Coupled Receptors
Source: Int J Mol Sci. 2020 Dec 27;22(1):187. doi: 10.3390/ijms22010187 (PMC7795036; doi:10.3390/ijms22010187)
Supplement: Supplementary file 1 [file ijms-22-00187-s001.zip › Supplementary materials and data/Supplementary materials.pdf]

Dynamical Correlations Reveal Allosteric Sites in G Protein-Coupled Receptors

Pedro Renault and Jesús Giraldo

Supporting Information

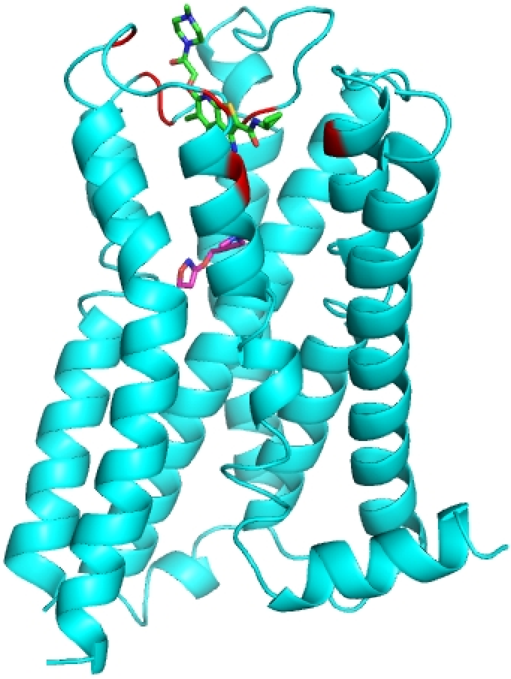

Figure S1. The M2 muscarinic receptor bound to the agonist iperoxo (purple sticks) and the allosteric modulator LY2119620 (green sticks) (PDB code 4MQT). Residues within 5 Å of the allosteric modulator are highlighted in red.

Table S1. PDB codes of structures in the experimental ensemble of β2AR

Inactive	Active
2RH1 [31], 3D4S [56], 3NY8, 3NY9, 3NYA [75], 3PDS [76], 4GBR [77], 5D5A, 5D5B [78], 5D6L [79], 5JQH [80], 6PRZ, 6PS0, 6PS1, 6PS2, 6PS3, 6PS4, 6PS5, 6PS6 [81]	3POG [82], 3SN6 [83], 4LDE, 4LDL, 4LDO [32], 4QKX [84], 6E67 [85], 6MXT [86], 6NI3 [87]

Numbers in brackets refer to the references in the main text.

Table S2. Residues in β2AR sites

Site	Residues
1	M40, I43, H93, I94, K97, M98, K305, E306, I309, L310, W313
2	I43, A46, I47, G50, L53, V54, V317, G320, F321, P323, L324, C327, F336
3	M215, V216, Y219, S220, F223, G276, M279, G280, T283
4	L64, T66, V67, T68, N69, I72, R131, Y141, Q142, S143, K147, R228, K270, A271, T274, L275, Y326, R328, S329, D331, F332
5	T164, P168, W173, Y199, A202, S203, V206
6	I121, E122, C125, V126, M156, V157, V160, V206, V210, P211
7	F71, V126, I127, V129, D130, R131, Y132, F133, A134, T136, S137, Q142, S143, L144, L145, V152
8	I55, I58, A59, L64, Q65, T66, N69, Y70, T73, S74, C77, R151, I154
9	A176, H178, E180, D192, F193, F194, T195, N196, Q197, H296, D300, N301, I303, R304, K305

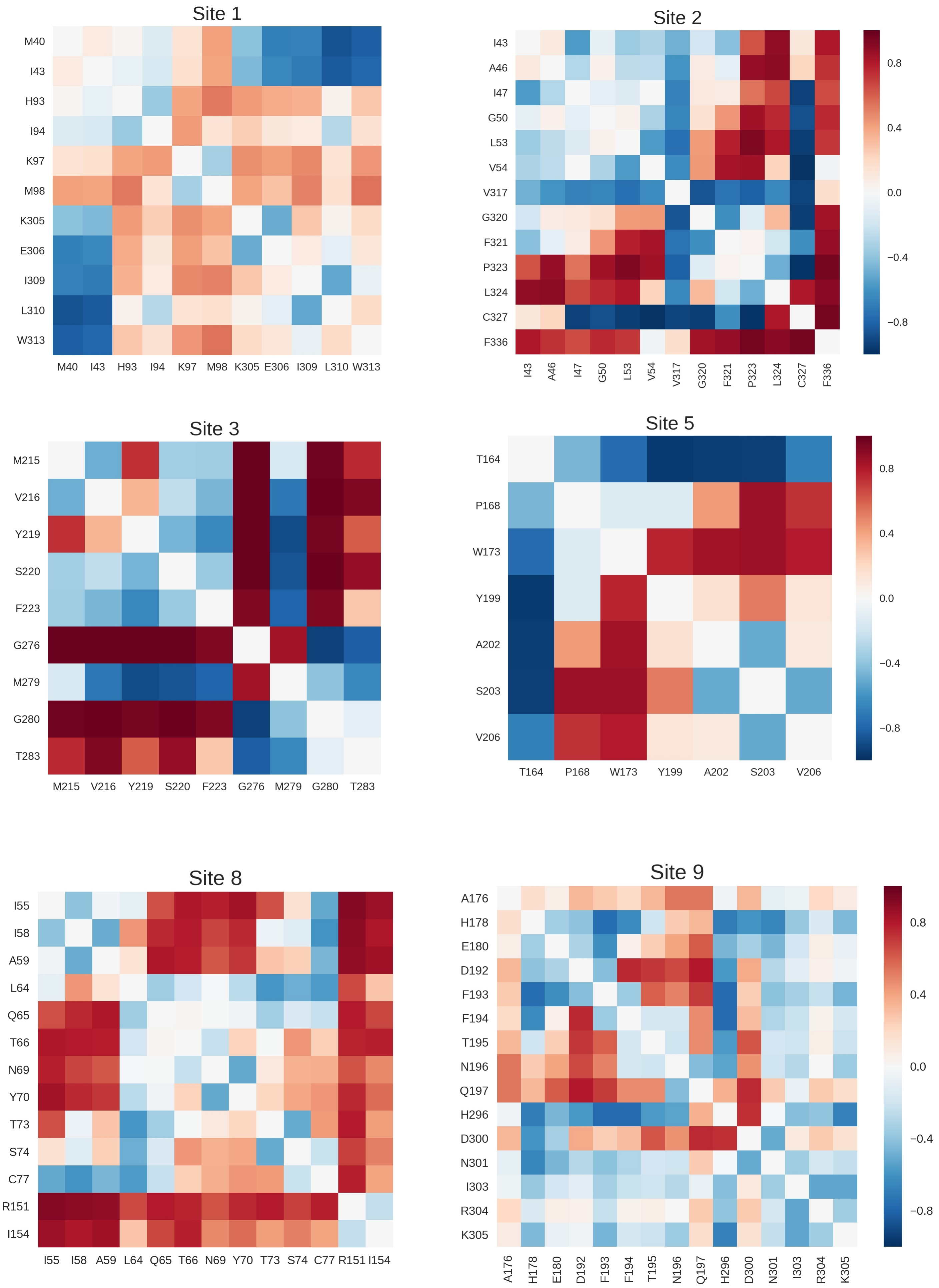
Site 1
M40 I43 H93 I94 K97 M98 K305 E306 I309 L310 W313
Site 2
I43 A46 I47 G50 L53 V54 V317 G320 F321 P323 L324 C327 F336
0.8 0.4 0.0 −0.4 −0.8
Site 3
M215 V216 Y219 S220 F223 G276 M279 G280 T283
Site 5
T164 P168 W173 Y199 A202 S203 V206
0.8 0.4 0.0 −0.4 −0.8
Site 8
I55 I58 A59 L64 Q65 T66 N69 Y70 T73 S74 C77 R151 I154
Site 9
A176 H178 E180 D192 F193 F194 T195 N196 Q197 H296 D300 N301 I303 R304 K305
0.8 0.4 0.0 −0.4 −0.8

Figure S2. **Matrices of correlations between inter-residue distances and Δ for sites in the β2AR.** Each element c_{ij} in these matrices is the correlation coefficient between Δ and the distance between residues *i* and *j*. Strong anti-correlations are in blue, intermediate values in white and strong correlations in red.

Table S3. Ranking of β2AR sites based on the strength of their coupling to Δ, obtained from NMA of the inactive conformation

Site Label	Coupling with Δ (C_{site})
**4***	**0.22**
**8**	**0.18**
**3**	**0.16**
6*	0.13
5	0.11
2	0.10
7*	0.10
1	0.09
9	0.08

Sites with C_{site} above the mean of the ranking are highlighted in bold. Those marked with * overlap with known allosteric sites

Table S4. Ranking of β2AR sites based on the strength of their coupling to Δ, obtained from the concatenation of harmonic trajectories (resulting from NMA) of the inactive and active conformations

Site Label	Coupling with Δ (C_{site})
**4***	**0.79**
**7***	**0.67**
**3**	**0.66**
**9**	**0.65**
5	0.59
6*	0.57
8	0.54
2	0.51
1	0.41

Sites with C_{site} above the mean of the ranking are highlighted in bold. Those marked with * overlap with known allosteric sites

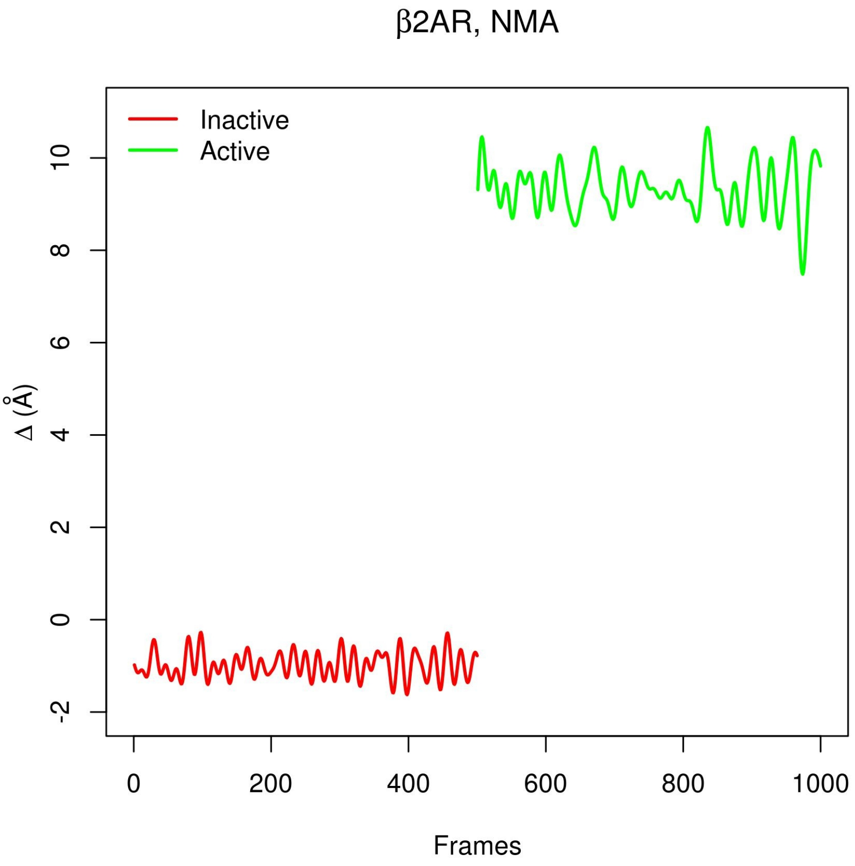

Figure S3. Variation of Δ in harmonic trajectories (resulting from normal mode analysis) of the β2AR

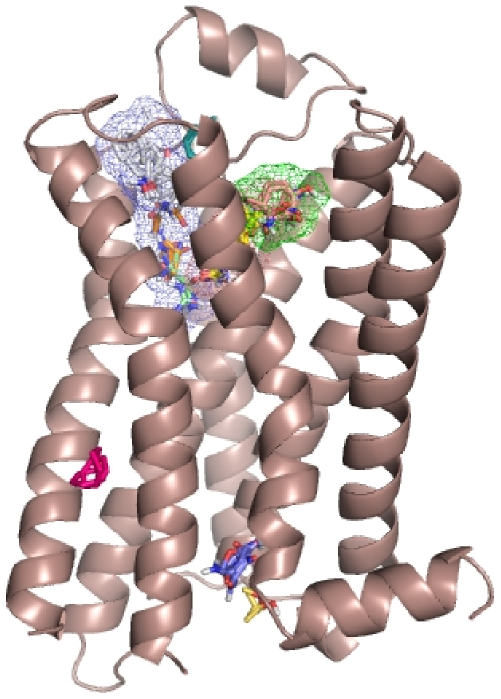

(a)

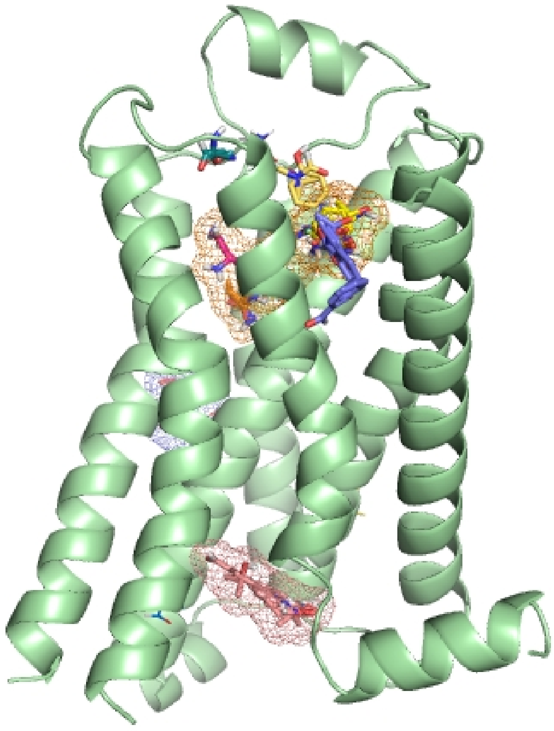

(b)

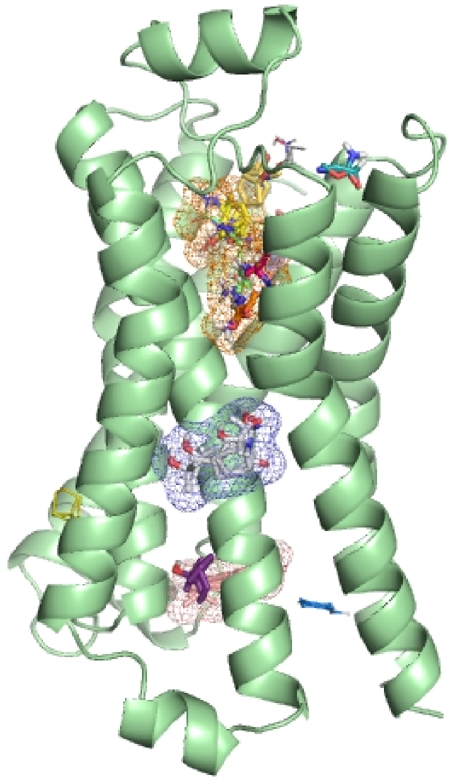

(c)

Figure S4. FTMap and FTSite results for the β2AR. FTMap probes are shown as sticks, and sites detected by FTMap are depicted in mesh representation. The stronger sites have the largest number of probes. A) Inactive conformation (PDB code 2RH1). FTMap identifies a total of 9 sites and the stronger ones are in the region of the orthosteric site. The region of the allosteric site identified by

us as Site 4, that overlaps the G Protein binding site, correponds to two FTMap sites, one ranked 4th (with 13 probes) and the other ranked 9th (with 3 probes). B) Active conformation (PDB code 4LDO). FTMap detects 14 sites in this case and the strongest ones are in the orthosteric site, and in the allosteric sites identified in our study as Site 4 (ranked 2nd, with 15 bound probes) and Site 6 (ranked 3rd, with 13 bound probes). The allosteric site corresponding to our Site 7, close to ICL2, is ranked 10th (with 3 bound probes).

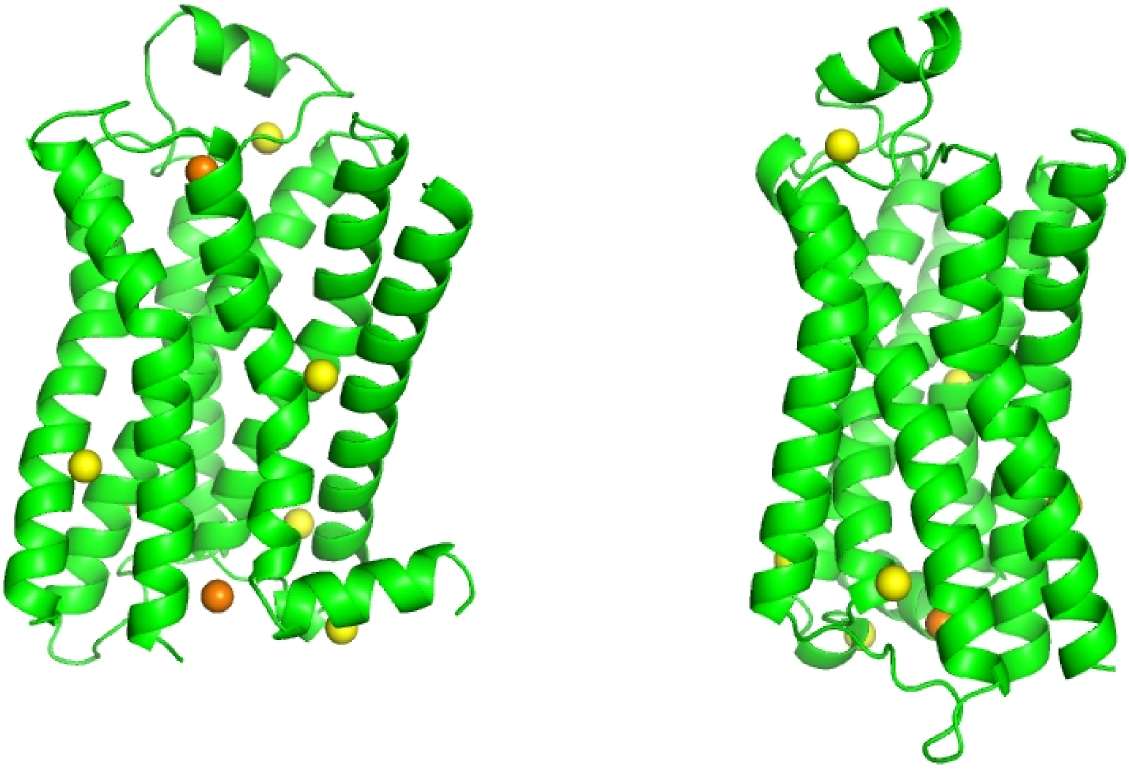

(a)

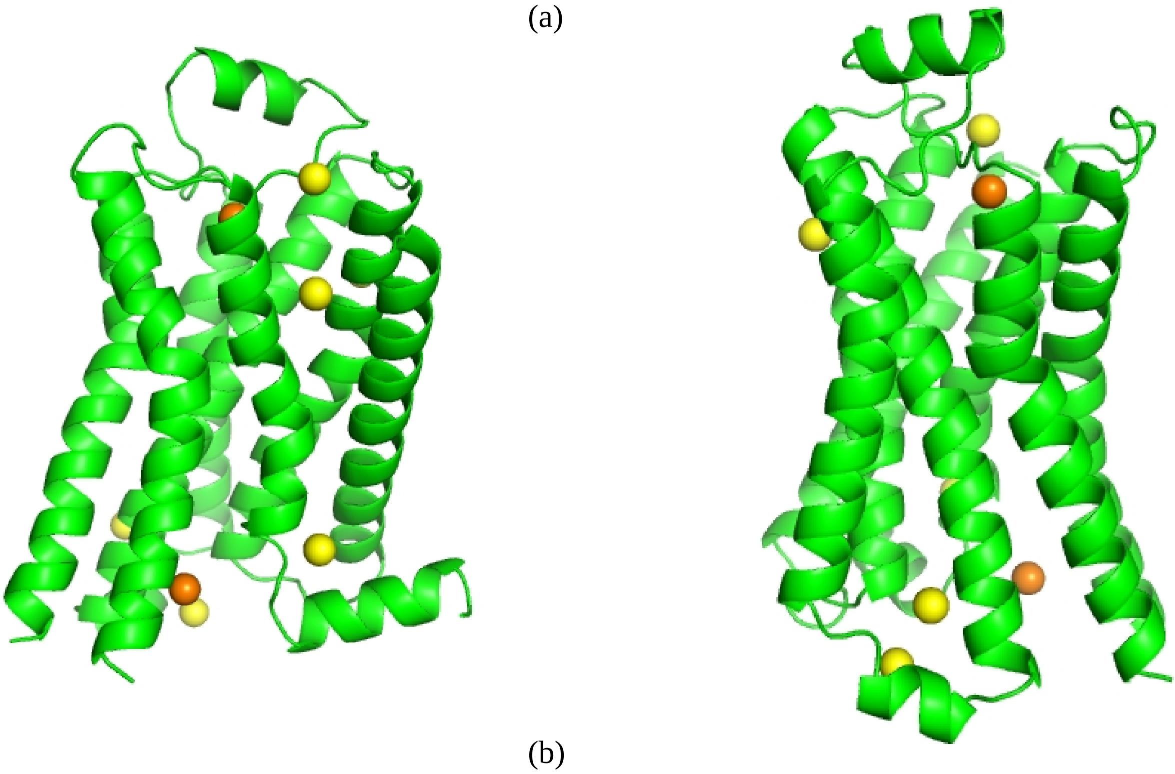

(b)

Figure S5. PARS results for the β2AR. PARS calculates p-values for the modified flexibility of

the protein upon perturbation of a site. Only sites resulting in p-values equal or less than 0.05 are considered to significantly alter the overall dynamics. Such sites are indicated by orange spheres, while, yellow spheres denote sites with higher p-values. A) Inactive conformation (PDB code 2RH1). Only cavities close to the orthosteric and G Protein binding sites were considered significant (p-values of 0.00 and 0.05, respectively). For the other cavities, the p-values varied between 0.25 and 0.83. B) Active conformation (PDB code 4LDO). Again, Only cavities close to the orthosteric and G Protein binding sites were considered significant (p-values of 0.03 in both cases). For the other cavities, the p-values varied between 0.15 and 0.72.

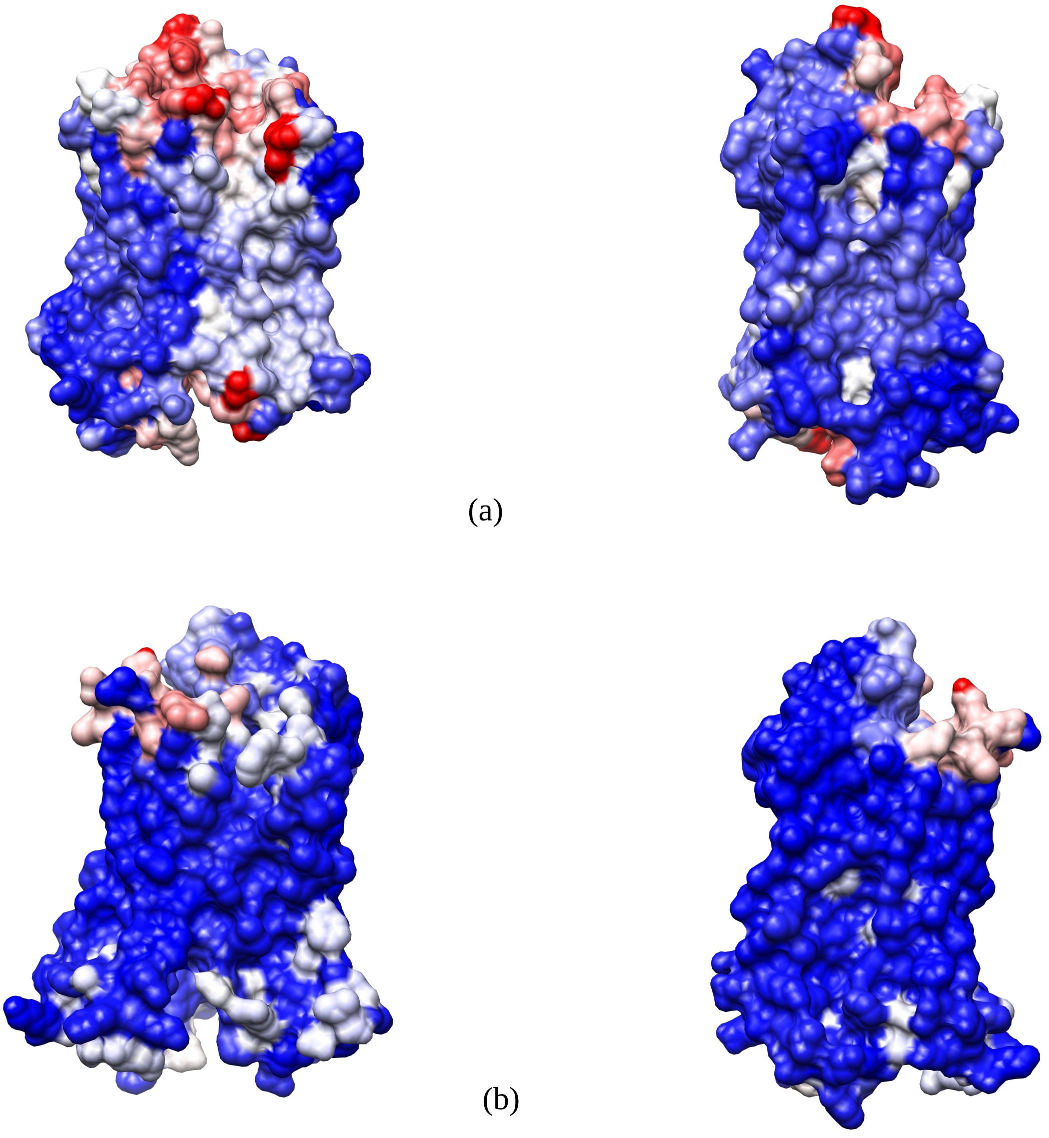

Figure S6. **SPACER results for the β2AR**. SPACER calculates the binding leverage to measure the coupling of a site to the global dynamics of the protein. The higher the binding leverage, the higher the probability of a site being allosteric. A) Inactive conformation (PDB code 2RH1). B) Active conformation (PDB code 4LDO). High binding leverage is depicted in red, intermediate values in white and low binding leverage is shown in blue. All calculations were performed with a probe peptide with 4 Cα's.

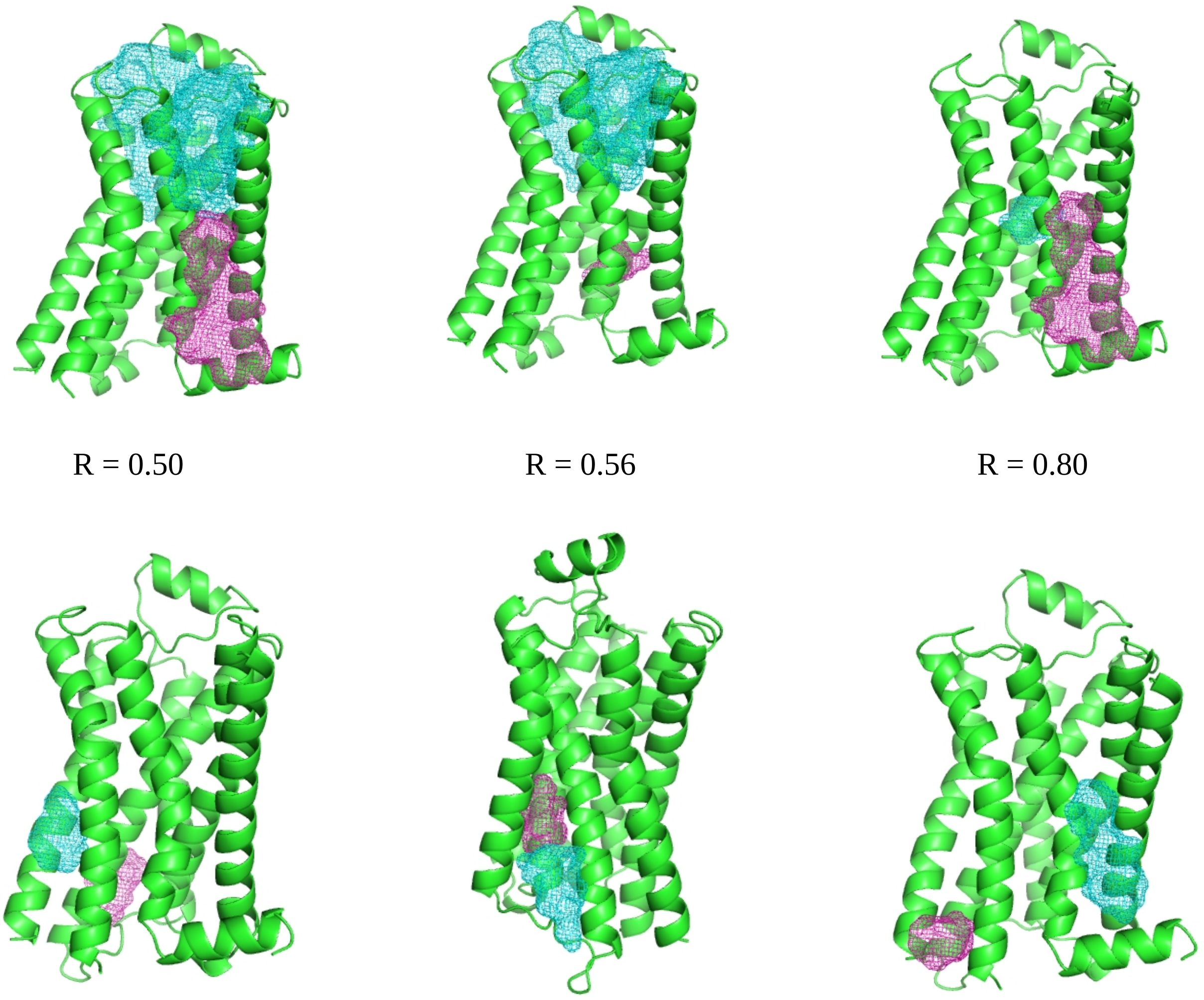

R = 0.50 R = 0.56 R = 0.80

R = 0.55 R = 0.54 R = 0.74

Figure S7. D3Pockets results for the β2AR. D3Pockets detects sites and calculates correlations between their volumes. The correlations between each pair of sites is shown below the illustrations, where sites are represented as mesh. Correlations were calculated across the experimental ensemble of β2AR.

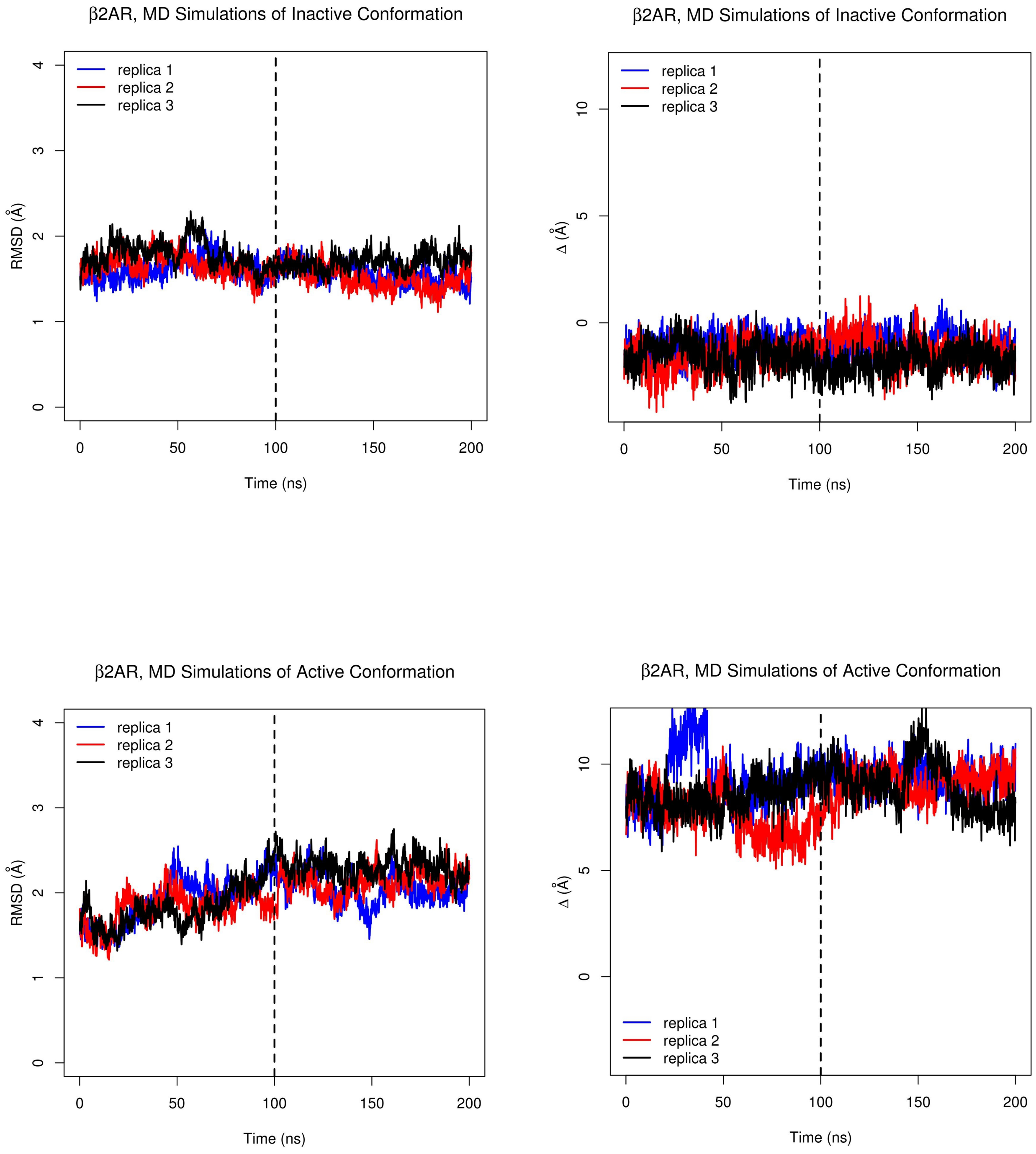

Figure S8. Time evolution of RMSD and Δ in MD simulations of the β2AR. Top: inactive conformation; bottom: active conformation. Left: RMSD; right: Δ. Graphics are shown for the three replicas in each conformational state. RMSD was measured on the backbone atoms of the receptor, with respect to the input crystal structure. Only the last 100 ns (at the right of the vertical dashed lined) were retained for the dynamical coupling analysis.

Table S5. Ranking of β2AR sites based on the strength of their coupling to Δ, obtained from the nine ensembles generated by molecular dynamics simulations. Values of C_{site} were calculated in each ensemble, and the table reports the mean value and the standard deviation (SD) for each site.

Site Label	Coupling with Δ (C_{site})
**4***	**0.65 (0.02)**
**2**	**0.54 (0.02)**
**7***	**0.50 (0.03)**
**1**	**0.46 (0.09)**
**3**	**0.45 (0.04)**
6*	0.39 (0.08)
9	0.36 (0.04)
5	0.33 (0.1)
8	0.33 (0.1)

Sites with C_{site} above the mean of the ranking are highlighted in bold. Those marked with * overlap with known allosteric sites.

Table S6. Residues in GCGR sites

Site	Residues
1	N300, W304, F312, V363, A366, F367, D370
2	V368, T369, E371, H372, L377, R378, K381
3	V236, C240, L243, I270, A274, P275, F278, F309, P310, L313, A314, I317
4	L277, F278, P281, W282, V285, K286, F289, E290, V292, W295, S297, D299, M301, G302, F303, W305, I306
5	Y239, L354, L357, L358, H361, F387, L388, S389, F391, Q392, G393, L395, V396
6	Y343, R346, L347, K349, S350, T351, L399, Y400, C401, L403, N404, K405
7	V185, L186, S189, S190, V193, V226, F230
8	L166, S167, K168, E410, R413, R414, R417
9	L253, L255, L328, L329, K332, L333, Q337, M338, H339, D342, K344, F345

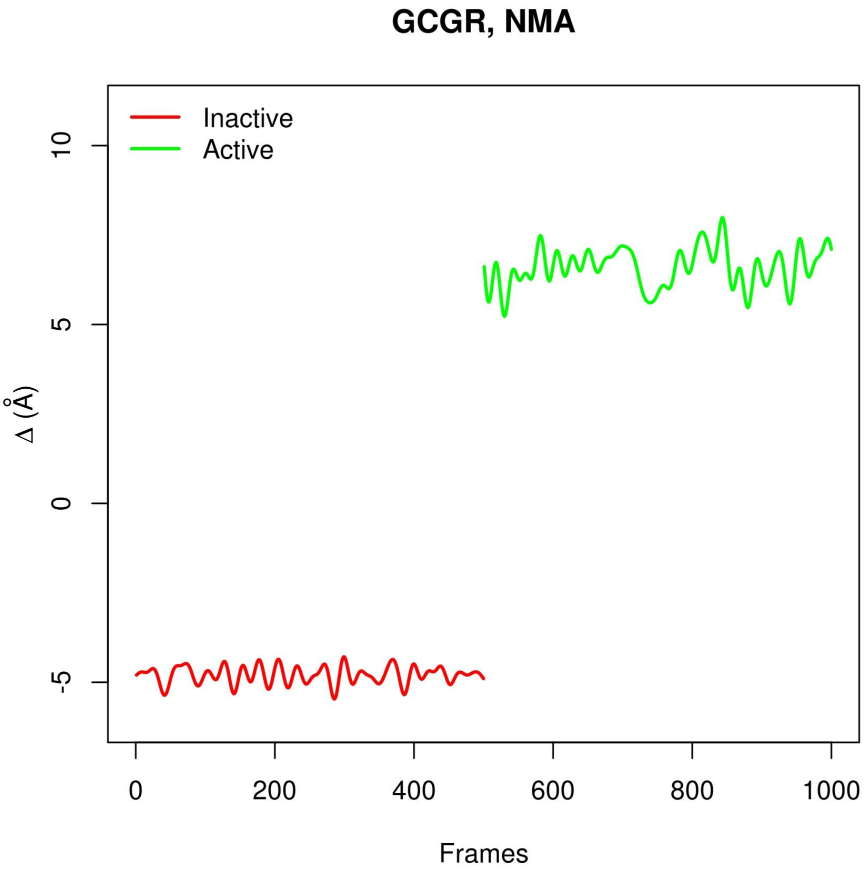

Figure S9. Variation of Δ in harmonic trajectories (resulting from NMA) of the GCGR

Table S7. Ranking of GCGR sites based on the strength of their coupling to Δ, obtained from NMA of the inactive conformation.

Site Label	Coupling with Δ (C_{site})
**6***	**0.65**
**5***	**0.54**
**3**	**0.50**
**4**	**0.46**
**9**	**0.45**
1	0.39
2	0.36
7	0.33
8	0.33

Sites with C_{site} above the mean of the ranking are highlighted in bold. Those marked with * overlap with known allosteric sites.

Table S8. Ranking of GCGR sites based on the strength of their coupling to Δ, obtained from the nine ensembles generated by molecular dynamics simulations. Values of C_{site} were calculated in each ensemble, and the table reports the mean value and the standard deviation (SD) for each site.

Site Label	Coupling with Δ (C_{site})
**1**	**0.61 (0.06)**
**6***	**0.59 (0.02)**
**4**	**0.55 (0.03)**
**9**	**0.51 (0.05)**
5*	0.48 (0.07)
3	0.45 (0.05)
8	0.40 (0.05)
2	0.39 (0.03)
7	0.36 (0.07)

Sites with C_{site} above the mean of the ranking are highlighted in bold. Those marked with * overlap with known allosteric sites.

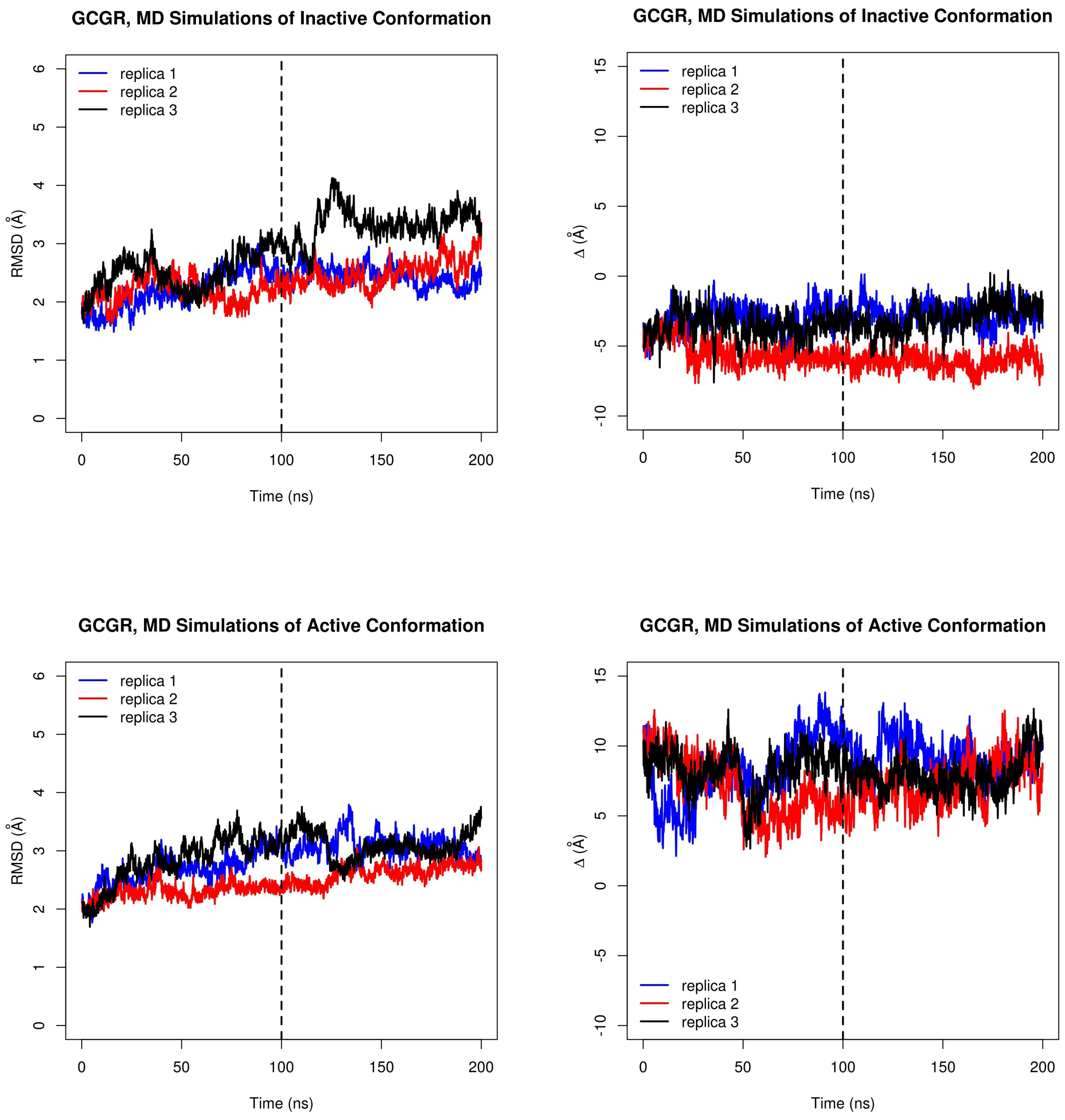

Figure S10. Time evolution of RMSD and Δ in MD simulations of the GCGR. Top: inactive conformation; bottom: active conformation. Left: RMSD; right: Δ. Graphics are shown for the three replicas in each conformational state. RMSD was measured on the backbone atoms of the receptor, with respect to the input crystal structure. Only the last 100 ns (at the right of the vertical dashed lined) were retained for the dynamical coupling analysis.

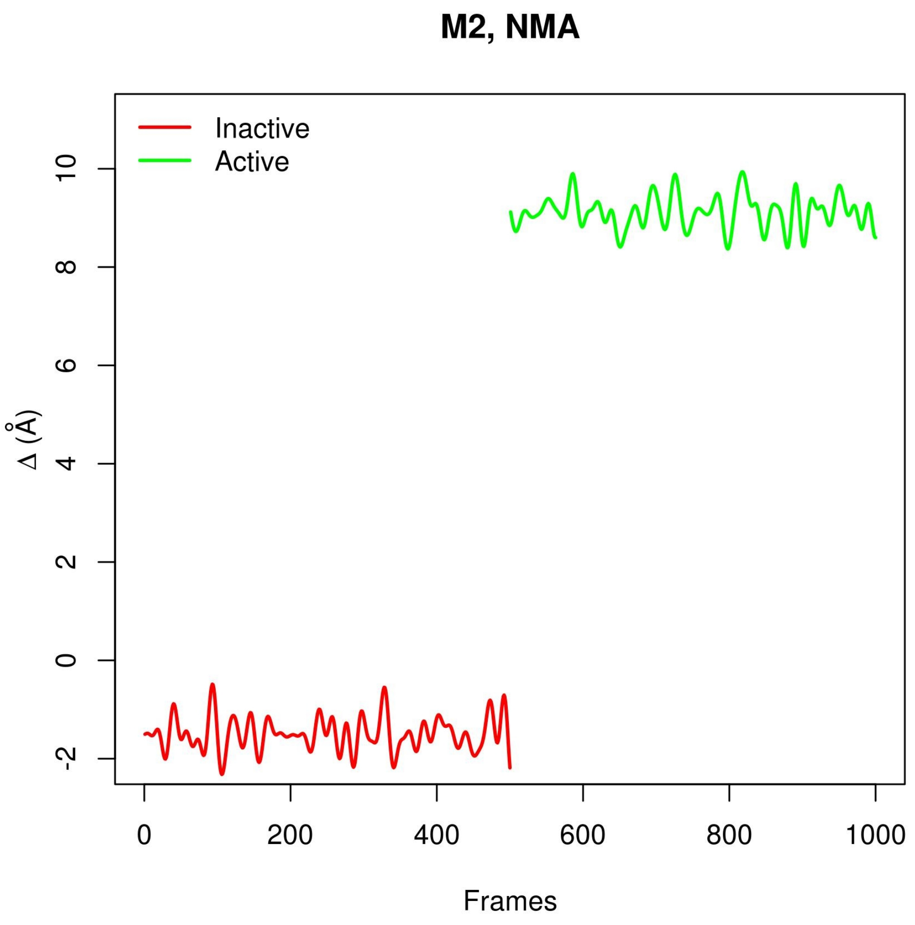

Figure S11. Variation of Δ in harmonic trajectories (resulting from NMA) of the M2 muscarinic receptor.

Table S9. Residues in M2 sites

Site	Residues
1	Y80, Y83, T84, G87, W89, W99, L100, D103, Y104, C176, Y177, I178, F181, T187, Y403, V407, N410, N419, W422, T423, Y426, Y430
2	F25, I26, V29, A30, L33, W427, I431
3	V36, I39, G40, L43, V44, T434, P437, A438, F451
4	L114, S118, F195, V199, M202, T203, Y206, L390, I392, L393, L394, F396, I397
5	N58, F61, L62, N113, L114, I117, S118, D120, R121, M202, Y206, T386, R387, T388, I389, I392, L393
6	N108, V111, M112, L154, W155, A158, I159, F180, G189, T190, A193
7	V57, F61, L115, I116, I117, F119, D120, F123, Y131, K134, R135, T136, M139, A140, M143
8	I48, K49, L54, Q55, T56, V57, N59, Y60, F63
9	V27, A30, G31, S34, M77, N78, T81, L82, V85, W427

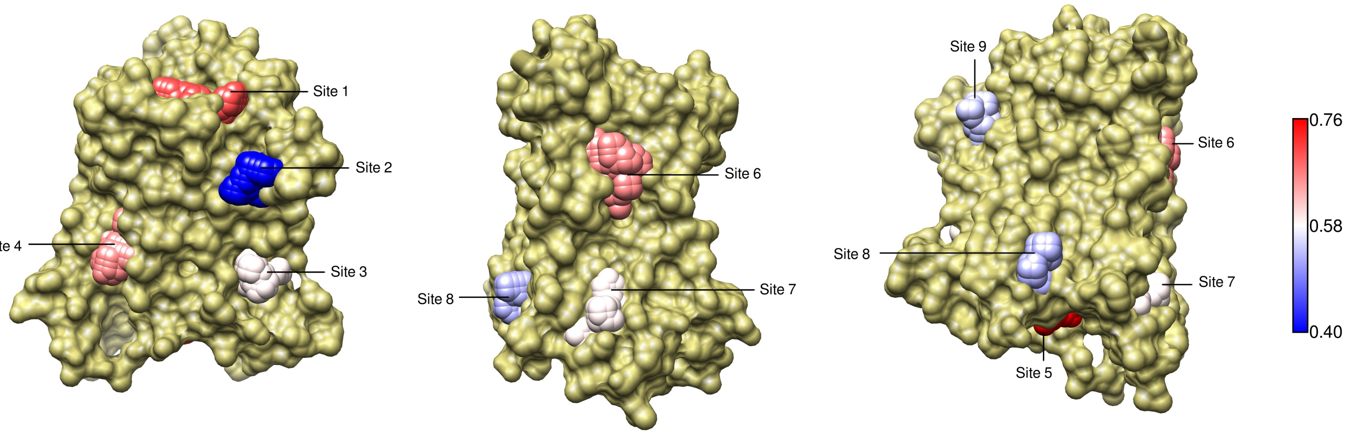

Figure S12. M2 sites couplings to Δ, obtained from the concatenation of harmonic trajectories (resulting from NMA) of the inactive and active conformations. Sites on the surface of M2 are represented by spheres, that are colored according to the coupling strength (from blue, denoting weaker coupling, to red, indicating stronger coupling). Site 1 corresponds to the allosteric site in PDB structure 4MQT.

Table S10. Ranking of M2 sites based on the strength of their coupling to Δ, obtained from the concatenation of harmonic trajectories (resulting from NMA) of the inactive and active conformations.

Site Label	Coupling with Δ (C_{site})
**5**	**0.76**
**1***	**0.72**
**4**	**0.69**
**6**	**0.69**
**3**	**0.64**
**7**	**0.64**
9	0.60
8	0.59
2	0.40

Sites with C_{site} above the mean of the ranking are highlighted in bold. The one marked with * overlaps with the known allosteric site.

Table S11. Ranking of M2 sites based on the strength of their coupling to Δ, obtained from NMA of the inactive conformation.

Site Label	Coupling with Δ (C_{site})
**5**	**0.27**
**4**	**0.21**
**3**	**0.16**
**8**	**0.14**
1*	0.12
7	0.09
6	0.08
2	0.07
9	0.07

Sites with C_{site} above the mean of the ranking are highlighted in bold. The one marked with * overlaps with the known allosteric site.

Table S12. PDB codes of structures in the experimental ensemble of the M2 muscarinic receptor

Inactive	Active
3UON [62], 5YC8, 5ZK3, 5ZK8, 5ZKB, 5ZKC [88]	4MQS, 4MQT [33], 6OIK [89], 6U1N [90]

Numbers in brackets refer to the references in the main text.

Table S13. Ranking of M2 sites based on the strength of their coupling to Δ, obtained from the ensemble of experimental structures

Site Label	Coupling with Δ (C_{site})
**5**	**0.66**
**1***	**0.63**
**7**	**0.60**
**6**	**0.58**
**4**	**0.53**
9	0.50
8	0.49
3	0.42
2	0.30

Sites with C_{site} above the mean of the ranking are highlighted in bold. The one marked with * overlaps with the known allosteric site.

Table S14. Ranking of M2 sites based on the strength of their coupling to Δ, obtained from the nine ensembles generated by molecular dynamics simulations. Values of C_{site} were calculated in each ensemble, and the table reports the mean value and the standard deviation (SD) for each site.

Site Label	Coupling with Δ (C_{site})
**5**	**0.58 (0.04)**
**6**	**0.50 (0.02)**
**4**	**0.41 (0.02)**
1*	0.35 (0.08)
7	0.35 (0.03)
3	0.33 (0.06)
9	0.27 (0.06)
2	0.24 (0.06)
8	0.24 (0.02)

Sites with C_{site} above the mean of the ranking are highlighted in bold. The one marked with * overlaps with the known allosteric site.

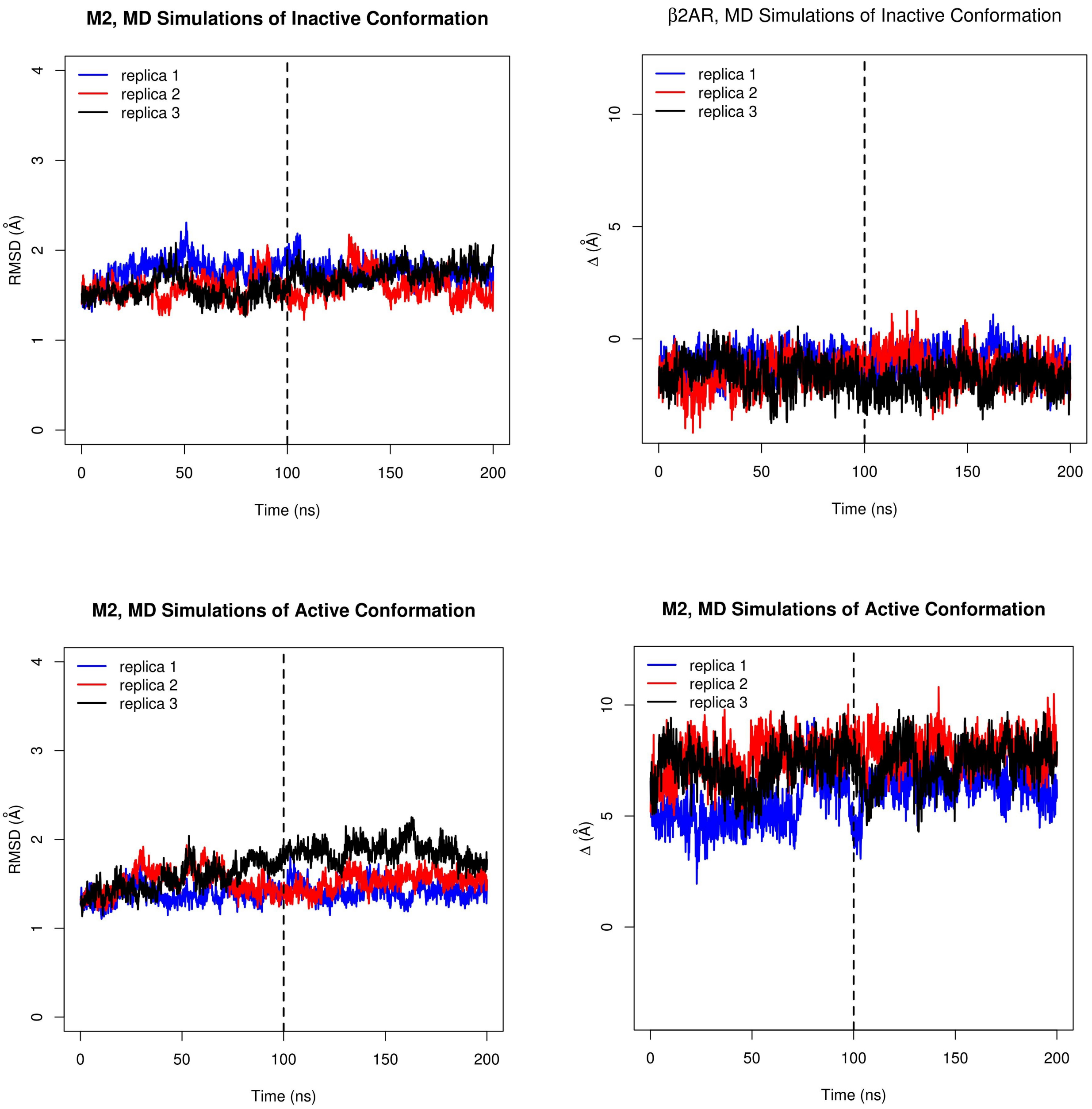

Figure S13. Time evolution of RMSD and Δ in MD simulations of the M2 muscarinic receptor. Top: inactive conformation; bottom: active conformation. Left: RMSD; right: Δ. Graphics are shown for the three replicas in each conformational state. RMSD was measured on the backbone atoms of the receptor, with respect to the input crystal structure. Only the last 100 ns (at the right of the vertical dashed lined) were retained for the dynamical coupling analysis.

Table S15. Summary of molecular dynamics simulations.

System	PDB input	State	Included residues	# atoms	# water molecules	# lipid molecules	orthosteric ligand	Nanobody
β2AR	2RH1 [31]	Inactive	32-230; 268-342	59071	11373	152	carazolol	--
β2AR	4LDO [32]	Active	28-231; 263-342	77661	16957	151	adrenaline	Nb6B9
GCGR	5XF1 [26]	Inactive	27-418	82243	18425	153	--	--
GCGR	6LMK [61]	Active	27-418	82717	18471	152	glucagon	--
M2	3UON [62]	Inactive	20-217; 377-456	58088	10939	154	QNB	--
M2	4MQS [33]	Active	19-215; 377-456	73003	15415	152	iperoxo	Nb9-8

Simulation time: 3 independent replicas of 200 ns for each system

Numbers in brackets refer to the references in the main text.

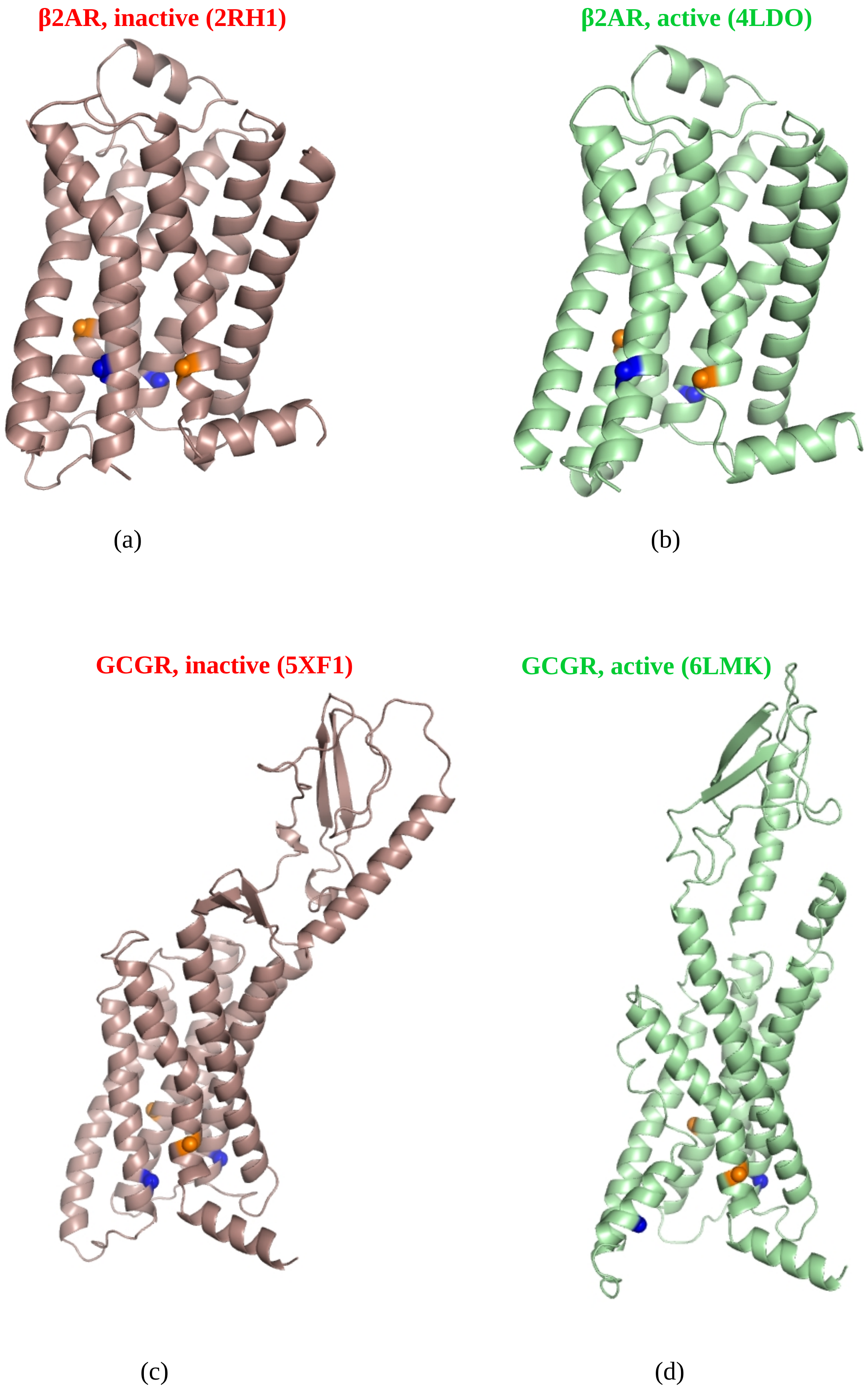
β2AR, inactive (2RH1)
β2AR, active (4LDO)
(a)
(b)
GCGR, inactive (5XF1)
GCGR, active (6LMK)
(c)
(d)

M2, inactive (3UON) **M2, active (4MQS)**

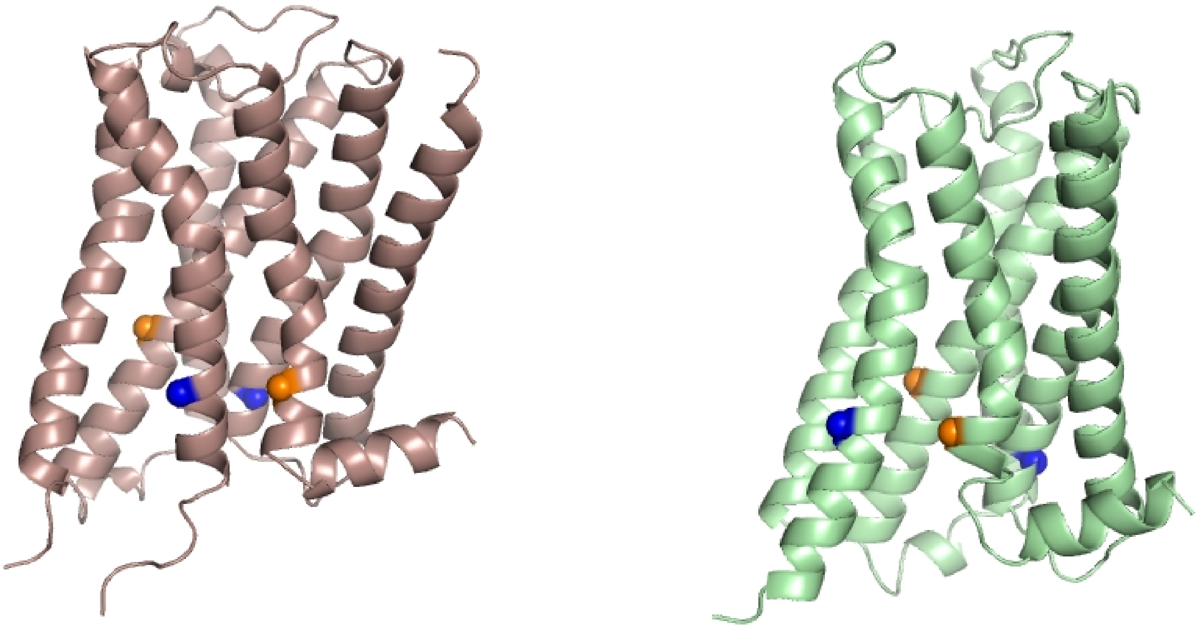

(e) (f)

Figure S14. Receptor structures used as inputs for NMA and MD simulations. Left, in red: inactive conformations; right, in green: active conformations. Cα's involved in the definition of Δ are shown as spheres. Atoms in TM2 and TM6 are shown in blue; atoms in TM3 and TM7 are shown in orange. Receptor models correspond to PDB structures a) 2RH1; b) 4LDO, c) 5XF1, d) 6LMK, e) 3UON and f) 4MQS.
